# Screening of *ent*-copalyl diphosphate synthase and metabolic engineering to achieve *de novo* biosynthesis of *ent*-copalol in *Saccharomyces cerevisiae*

**DOI:** 10.1016/j.synbio.2024.06.005

**Published:** 2024-06-18

**Authors:** Shan Li, Shuangshuang Luo, Xinran Yin, Xingying Zhao, Xuyang Wang, Song Gao, Sha Xu, Jian Lu, Jingwen Zhou

**Affiliations:** a Engineering Research Center of Ministry of Education on Food Synthetic Biotechnology, School of Biotechnology, Jiangnan University, 1800 Lihu Road, Wuxi, Jiangsu, 214122, China; b School of Biotechnology, Jiangnan University, 1800 Lihu Road, Wuxi, Jiangsu, 214122, China; c Science Center for Future Foods, Jiangnan University, 1800 Lihu Rd, Wuxi, Jiangsu, 214122, China

**Keywords:** *Andrographis paniculata*, *Ent*-copalyl diphosphate synthase, *ent-*CPP, Diterpene synthase, Acetyl-CoA, Quantification

## Abstract

The diterpene *ent*-copalol is an important precursor to the synthesis of andrographolide and is found only in green chiretta *(Andrographis paniculata)*. *De novo* biosynthesis of *ent*-copalol has not been reported, because the catalytic activity of *ent*-copalyl diphosphate synthase (CPS) is very low in microorganisms. In order to achieve the biosynthesis of *ent*-copalol, *Saccharomyces cerevisiae* was selected as the chassis strain, because its endogenous mevalonate pathway and dephosphorylases could provide natural promotion for the synthesis of *ent*-copalol. The strain capable of synthesizing diterpene geranylgeranyl pyrophosphate was constructed by strengthening the mevalonate pathway genes and weakening the competing pathway. Five full-length *Ap*CPSs were screened by transcriptome sequencing of *A. paniculata* and *Ap*CPS2 had the best activity and produced *ent*-CPP exclusively. The peak area of *ent*-copalol was increased after the *Ap*CPS2 saturation mutation and its configuration was determined by NMR and ESI-MS detection. By appropriately optimizing acetyl-CoA supply and fusion-expressing key enzymes, 35.6 mg/L *ent*-copalol was generated. In this study, *de novo* biosynthesis and identification of *ent*-copalol were achieved and the highest titer ever reported. It provides a platform strain for the further pathway analysis of andrographolide and derivatives and provides a reference for the synthesis of other pharmaceutical intermediates.

## 1 Introduction

*Andrographis paniculata* (Burm. f.) Nees, commonly known as creat or green chiretta, is an annual herbaceous plant in the family Acanthaceae [1]. It has remarkable antibacterial and anti-inflammatory effects and is also known as the Chinese medicinal antibiotic [2], being selected as an homology of medicine and food in Chinese herbal medicine and consumed as a nutritional food ingredient to maintain health. Andrographolide is an *ent-*labdane-related diterpene and is the main functional component of *A. paniculata* [3]. It has efficacy against viral diseases, such as new crown pneumonia, influenza and dengue fever [4]. The variation in andrographolide content of different varieties of *A. paniculata* is wide, ranging from 3.9 to 9.7 mg/g [5]. Though it has been proposed that the biosynthesis of andrographolide is initiated from specific *ent*-copalol, the enzymes that catalyzes the formation of *ent*-copalol from *ent*-CPP and the key genes for downstream reactions in *A. paniculata* have not been explored [6].

The *ent*-copalol is a unique precursor involved in the synthesis of andrographolides and is found only in *A. paniculata*. As an important precursor for the synthesis of andrographolide, the *ent*-coaplol is biosynthesized in high quantities in *S. cerevisiae* which is of great significance for the analysis of the synthetic pathway of andrographolide. It is the product of *ent*-copalyl diphosphate dephosphorylation (*ent*-CPP) and a unique precursor of andrographolide. Geranylgeranyl pyrophosphate (GGPP) is a universal precursor of diterpenoids and is also andrographolide, which is converted from isopentenyl pyrophosphate (IPP) and dimethylallyl pyrophosphate (DMAPP) as basic units [7,8]. This GGPP was catalyzed by the Class II diterpene synthase *ent*-copalyl diphosphate synthase (CPS) to form *ent*-CPP with a parental loop structure [9]. The *ent*-copalol could be detected by screening for heterologous expression of CPS in *Escherichia coli* (*E. coli*) [10], but the catalytic activity of the screened CPS was low and the *ent*-copalol lacked commercial standards and could not be quantified, which hindered the analysis of the downstream pathway of andrographolide.

Due to the presence of the endogenous MVA pathway, *S. cerevisiae* is widely used in the study of terpenoid synthesis [11] as it has a relatively strong supply capacity of mevalonate precursors and has a better post-translational modification system of endoplasmic reticulum, golgi apparatus and other inner membrane structures [12]. Enhancement of the MVA pathway and modification of the acetyl-CoA pathway has been confirmed to play an important role in the synthesis of diterpenes, which can significantly improve the yield of the product^13^*.* In particular, *S. cerevisiae* contains isoprene-pyrophosphate phosphatase (*DPP1/LPP1*) which promotes the conversion of the *ent*-CPP to the parent nucleus structure *ent*-copalol [14], so it is an excellent host for the synthesis of *ent*-copalol. This study investigated how to promote the transformation of GGPP to *ent*-copalol and reduce the loss of competitive pathway are the key issues to increase the titer of *ent*-copalol.

**Fig. 1:**
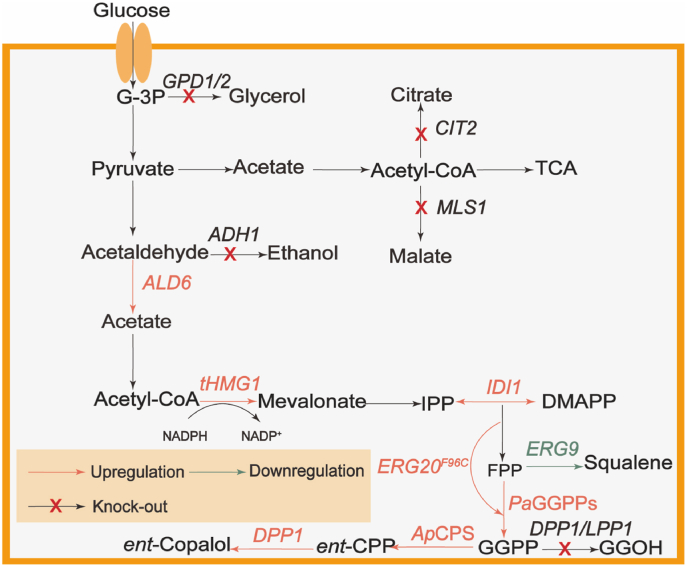
*De novo* biosynthesis of *ent*-copalol in *S. cerevisiae*. *ALD6*, acetaldehyde dehydrogenase; *CIT2*, peroxisomal citrate synthase; *ADH*, alcohol dehydrogenase; *GPD*, glycerol 3-phosphate dehydrogenase; *Pa*GGPPs, GGPP synthetase from *Pantothecia agglomerata*; *ERG9*, squalene synthase; *MLS1*, Malate synthase; *Ap*CPS, *ent-*copalyl diphosphate synthase from *Andrographis paniculata*; *DPP1*, Diacylglycerol pyrophosphate (DGPP) phosphatase.

To obtain more comprehensive transcriptome information, various tissues of *A. paniculata* were analyzed by RNA-seq technology. The diterpenoid synthetic chassis strain was constructed by strengthening the mevalonate pathway. The five *Ap*CPS enzymes were further identified and *Ap*CPS2 which could be highly expressed was screened. The synthesis pathway of *ent*-kaurinonic acid was introduced to indirectly confirm the production of *ent*-CPP. After *Ap*CPS2 saturation mutation, the strain with the largest increase in peak area was cultured for 96 h, then the culture broth was purified and pure *ent*-copalol was identified. The metabolic pathway was modified and the competition pathway was inhibited, seen in Fig. 1 and CW10071 then *de novo* biosynthesized 35.6 mg/L *ent*-copalol in shake flasks. The titer of *ent*-copalol was quantified, providing a platform strain for the heterologous synthesis of andrographolide from *S. cerevisiae*.

## 2 Materials and methods

### 2.1 Strains, plasmids, and chemicals

The sequences of the synthetic genes and the screened genes in this study are shown in Table S1 and include Diterpene synthase (Gene bank: MW343729) from *Pantoea agglomerans* (*Pa*GGPPS); Diterpene synthase (Gene bank: QUF98547.1) from *Taxus media* (*Tm*GGPPs); *ent*-kaurene synthase (GenBank: AY347877.1) from *Oryza sativa* (*Os*KS); *ent*-kaurene oxidase (Gene bank: AAQ63464.1) from *Stevia. Rebaudiana (Sr*KO*)* and *ent*-kaurene synthase-like (Gene bank: BE585476) from *Triticum aestivum* (*Ta*KSL1).

Plant-derived genes are difficult to express in *S. cerevisiae*, so the heterologous genes were codon optimized and synthesized by GENCEFE Biotech Co., Ltd (Wuxi, China) according to the codon preference of *S. cerevisiae*. All fragments were obtained by PCR; gel, or column purification was performed using the DNA Extraction Mini Kit (Vazyme Biotech Co., Ltd, Nanjing, China). The engineered strain C800 (gal80KanMX) derived from *S. cerevisiae* CEN.PK2-1D, was used as the starting strain [15]. The *E. coli* JM109 was used for plasmid construction and storage and *E. coli* BL21(DE3) was used to express proteins. The plasmid pET-28a (+) was used as the *Ap*CPSs expression vector.

### 2.2 The CRISPR experiments

The CRISPR-Cas9 system was used to integrate genes into *S. cerevisiae* [16]. The plasmid p414-TEF1p-Cas9-CYC1t was constructed previously [16]. The guide RNAs were designed by the online software Yeastriction (http://yeastriction.tnw.tudelft.nl/). The plasmid pRS423 with *His* was used to express the target gene [17] and all plasmids were constructed using Gibson assembly [18] and confirmed by Sanger sequencing before yeast transformation. Details of plasmids used in this study are shown in Table S2 and all primers used in this study are shown in Table S3. The lithium acetate method was used for *S. cerevisiae* transformation [19]. All chemical reagents were purchased from Sangon Biotech Co., Ltd (Shanghai, China).

### 2.3 RNASeq analysis of *A. paniculata* tissues

The *A. paniculata* plants were purchased in April 2020 at the Chinese medicinal materials base in Nanning, Guangxi and grown in a climatic incubator (Youfeng Scientific Instrument Biotech Co., Ltd, Shanghai, China) until the flowering period, with a light intensity of 2 lux and at a temperature of 25 °C.

Spires, roots, stems, flowers and cotyledons of three different plants were sampled, with three biological replicates per tissue. Dead leaves and parts damaged by insects were removed. The remaining tissues were washed gently with ultrapure water, wiped clean, then 0.2–0.5 g of each part was placed into a 5 mL centrifuge tube and snap-frozen in liquid nitrogen.

### 2.4 Gene amplification and analysis

The cDNA was obtained with gDNA eraser reverse transcription reagent [20], from the PrimeScript RT reagent kit (Takara Biotech Co., Ltd, Beijing, China). The target genes were amplified according to the RNASeq database which was extracted by Wuhan Hope Group Biotech Co., Ltd (Wuhan, China), with the amplification primers listed in Table S3. Biological evolutionary trees were constructed using Mega software version 7.0 (Table S4). The Alphafold2 tool (https://colab.research.google.com/) was used for homology modeling. Docking simulations were performed by BIOVIA Discovery Studio (Version 2019). AutoDock Vina was used for molecular docking version 1.2.0. PyMol software version 2.5.2 was used for visual analysis of protein structure [18].

### 2.5 Strain culture conditions

Luria bertani (LB) medium made up of 10 g/L peptone, 5 g/L yeast extract and 10 g/L sodium chloride was used for culturing *E. coli* and 100 mg/L ampicillin was added where necessary. Yeast extract peptone dextrose (YPD) medium made up of 1 % yeast extract, 2 % peptone and 2 % anhydrous glucose was used for activation and culture of *S. cerevisiae* strains and 1.5 % agar powder was added to produce solid YPD and LB media [21].

The engineered *S. cerevisiae* strains were grown on synthetic dropout medium containing 1.74 g/L yeast nitrogen base (YNB) without amino acids, 20 g/L glucose and 5 g/L (NH_4_)_2_SO_4_ and supplemented with 50 mg/L uracil and 50 g/L of appropriate amino acids including histidine, tryptophan and leucine, depending on the auxotroph of the strain, with 15 g/L agar added as required.

For shake flask fermentation, recombinant yeast was inoculated onto YNB solid plates, activated at 30 °C, then a single colony was transferred into 5 mL liquid YNB medium at 30 °C and activated at 200 rpm for 20 h to form the seed solution. Finally, 25 mL liquid YPD medium was inoculated and incubated for 96 h according to the initial optical density at 600 nm (OD_600_) = 0.1of *S. cerevisiae* [15].

### 2.6 Expression and purification of *Ap*CPSs

*The Ap*CPS1-5 was assembled with vector pET-28a(+) to form plasmid pET28a-1 to 5 and these plasmids were transformed into E. coli BL21(DE3) competent cells. A single colony was inoculated into LB medium with 100 mg/L ampicillin and cultured at 37 °C overnight. The cultures were then inoculated into 250 mL of terrific broth (TB) medium with 100 mg/L ampicillin and grown at 37 °C. The protein expression was induced with 0.1 mM isopropyl-β-*d*-thiogalactopyranoside (IPTG) at 20 °C for 20 h when the OD_600_ was 0.6–0.8.

Cells were collected by centrifugation and resuspended in PBS in a buffer ultrasonic cell crusher noise isolating chamber. After the ultrasonic cell disruptor was disrupted, the supernatant was collected by centrifugation and the purified protein was analyzed with sodium dodecyl sulfate polyacrylamide gel electrophoresis (SDS-PAGE).

### 2.7 Extraction of active ingredients from plant tissues and culture media

Various *A. paniculata* tissues were sampled at the flowering stage, washed and dried and transferred to 10 mL centrifuge tubes. 8 mL methanol and 2 mL *n*-hexane were added and the samples were macerated for 1 h, sonicated for 30 min, then centrifuged at 4000×*g* for 10 min. The *n*-hexane layer was used to analyze *ent*-copalol by gas chromatography-mass spectrometry (GC-MS), the methanol layer was used to analyze andrographolide by high performance liquid chromatography (HPLC) and the plant tissue precipitation was discarded.

Intracellular *ent*-copalol was detected by centrifuging the cells in the culture broth. One mL of *n*-hexane was then added to the crushing tube and cells were lysed under high pressure in a FastPrep homogenizer (MP Biomedicals Co., Ltd., Illkirch-Graffenstaden, France). To separate the supernatant, the lysed cell solution was centrifuged at 5000 *g* for 5 min. Culture broth and *n*-hexane (1:1) were added into the centrifuge tube, shaken for 3 min, centrifuged at 4000×*g* for 2 min and the organic phase was taken to measure the content of extracellular products.

### 2.8 Gas Chromatography–Mass spectrometry (GC-MS)

The HPLC used a 5 μm, 4.6 mm × 250 mm C18 column (Thermo Fisher Scientific, Inc., Waltham, MA, USA), with the mobile phase A as water and B as methanol. Analytes were eluted with a linear gradient, initially 30 % B, increasing to 60 % B at 12 min, maintained until 20 min, decreased to 30 % B at 23 min and maintained until 30 min. The flow rate was 1 mL/min and the injection volume was 10 μL.

The GC-MS detection used an electronic bombardment source with a bombardment voltage of 70 eV, a scan range of 50–400 amu *m*/*z* and an ion source temperature of 230 °C. A 30 mm × 0.35 mm × 0.25 μm HP-5MS capillary column was used with an inlet temperature of 280 °C, column temperature of 70 °C for 2 min, increasing by 10 °C/min to 280 °C, where it was held for 2 min, with an acquisition delay of 10 min. The carrier gas was helium, the flow rate was 1 mL/min and the injection volume was 1 μL.

### 2.9 Nuclear magnetic resonance (NMR) analysis

The HPLC-ESI-MS system (Thermo Fisher Scientific) was equipped with a 5 μm, 4.6 mm × 150 mm cosmosil 5C_18_-MS-II column. The mobile phase A was water with 0.1 % phosphoric acid, mobile phase B was acetonitrile, the temperature was 35 °C and the flow was 0.3 mL/min^18^. The conditions of isocratic elution for MS were electrospray ionization (ESI) negative, source block temperature of 110 °C, desolvation temperature of 400 °C, mass range of 20–2000 *m*/*z* and detector voltage of 1800 V.

Samples were purified using an LC-20AR semi-preparative chromatography system using a 10 mm × 250 mm, 5 μm Shim-pack GIST C18 column (Shimadzu, Japan) at 210 nm and 40 °C. The elution program consisted of acetonitrile and water (35:70) at a flow rate of 5 mL/min and elution for 50 min. The purified samples were dissolved in CDCl_3_ and subsequently characterized by NMR spectra with an Avance III 600 MHz nuclear magnetic resonance spectrometer (Bruker BioSpin, Karlsruhe, Germany). The NMR spectra were recorded on 500 MHz for ^1^H and 126 MHz for ^13^C in CDCl_3_^22^. MestReNova software 14.0 was used to analyze and process data.

## 3 Results

### 3.1 Distribution of *ent*-copalol in *A. paniculata* and transcriptome analysis

**Fig. 2:**
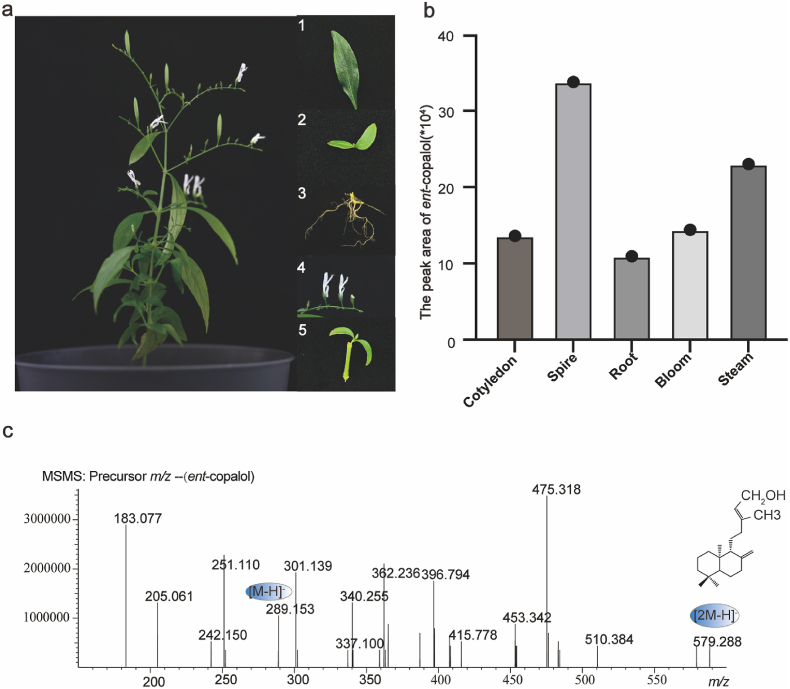
Evaluation of *A. paniculata*. (**a**) *A. paniculata* plant with close-up images of the cotyledon, spire, root, bloom and stem. (**b**) The content of *ent*-copalol in *A. paniculata*. (**c**) Mass spectrum of *ent*-copalol.

To understand the distribution of *ent*-copalol in *A. paniculata*, the composition of andrographolide from different tissues was examined (Fig. 2a). The *ent*-copalol with *m/z* of 290 was faintly detected in plant tissues (Fig. 2b and c), which may be due to the direct precursor *ent*-CPP of *ent*-copalol involved in multiple metabolic pathways in Fig. S1 and the efficient catalysis of *ent*-copalol to andrographolide production by enzymes *in vivo*. The result was the same distribution of components as reported in the literature [23]. The *ent*-copalol and andrographolide were found only in the tissues of *A. paniculata*, so bloom, spire, cotyledon, stem and root were selected to send to a third-party platform for RNA-Seq.

After RNA-Seq detection in the samples, 752,562 congruent sequences were obtained in the first cluster, with a total length of 1,939,920,567 bp and an average length of 2577 bp. The second clustering of IsoSeq3 yielded 364,424 congruence sequences and the total length of the congruent sequences corrected by second-generation data was 948,013,621 bp and the average length was 2601 bp. After CD-hit de-redundancy, 285,708 non-redundant transcript sequences with an average length of 2625 bp and 73,962 unigene sequences with an average length of 2820 bp were obtained. Through the KOG, KEGG, NR, Swissprot and GO databases, a total of 72,717 genes were annotated in 73,962 genes, with an annotation rate of 98.32 % (Fig. S2). The results of this assay provide a new opportunity to identify all CPS genes involved in andrographolide biosynthesis.

### 3.2 Construction of diterpene platform strains and screening of diterpene synthases

**Fig. 3:**
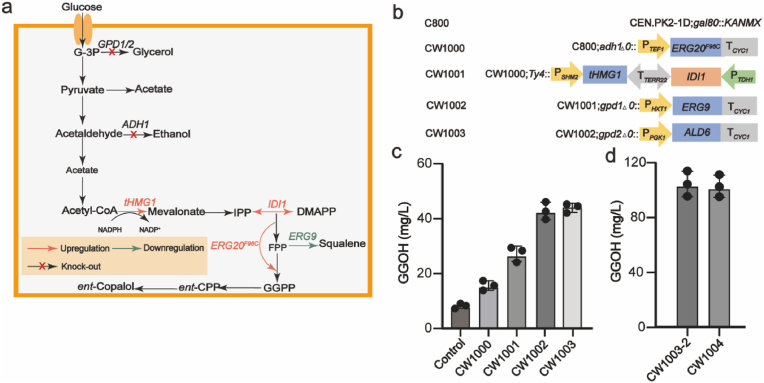
*De novo* biosynthesis of GGOH in *S. cerevisiae*. (**a**) Summary of metabolic engineering modifications in *S. cerevisiae* strain CW1003, designed to increase production of precursor GGPP by enhancing the MVA pathway and inhibiting competing pathways. (**b**) Construction of strain CW1003. (**c**) Titer of GGOH after 96 h fermentation for screening GGPPs. (**d**) *Pa*GGPPs are integrated on the genome to form the titer of GGOH 96 h after fermentation of CW1004.

To screen the key genes involved in the biosynthesis of *ent*-copalol from *A. paniculata* in *S. cerevisiae*, a strain was constructed for *de novo* biosynthesis of GGPP. The research confirmed that the MVA pathway metabolism was enhanced by reinforcing genes *tHMG1* and *IDI1* and mutating *ERG20* to *ERG20*^*F96C*^ [24], by-product ethanol and glycerol production was reduced by knocking out *ADH1*, *GPD1* and *GPD2* [25] and the catalytic conversion of farnesyl pyrophosphate (FPP) to squalene was reduced by replacing the original promoter of *ERG9* with HXT1p [26] (Fig. 3a). The modification strategies of the above genes integrated directly into the genome to form the reconstituted *S. cerevisiae* chassis strain CW1000 to CW1003 and GGPP flux was highest in CW1003 with geranylgeraniol (GGOH) reaching 48.6 mg/L (Fig. 3b and c).

Three GGPP synthases were screened from *A. paniculata*. Each of GGPPs including *Tm*GGPPs, *Pa*GGPPs [27], *Ap*GGPPs1, *Ap*GGPPs2 and *Ap*GGPPs3 were constructed on the pRS423 vector to form five plasmids [13,28] and were transformed into CW1003 to form strains CW1003-1 to CW1003-5, respectively. The strain CW1003-2 overexpressing *Pa*GGPPs had the highest GGOH yield of 107.0 mg/L in Fig. S3. The *Pa*GGPP which could improve the conversion efficiency of FPP to GGPP was integrated on into the genome of *S. cerevisiae* CW1003, resulting in strain CW1004, which produced the titer of GGOH was consistent with plasmid expression (Fig. 3d). As a result, CW1004 was used as a chassis strain for subsequent screening of *Ap*CPS.

### 3.3 Screening *Ap*CPS and determining the CPP stereoconfiguration

**Fig. 4:**
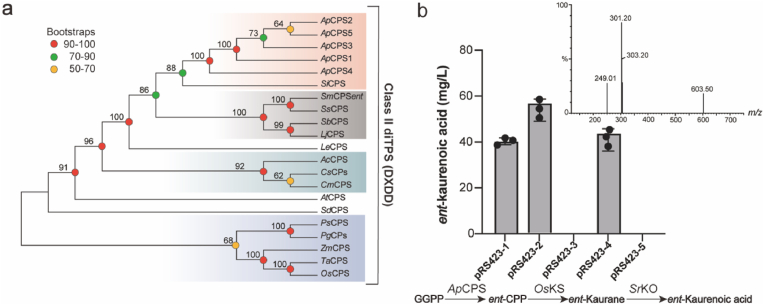
Evolutionary relationship and functional characterization of *Ap*CPS. (**a**) Evolutionary tree of *Ap*CPS and *ent*-copalyl diphosphate synthase that has been functionally characterized. (**b**) Titer of *ent*-kaurenoic acid after introduction of five *Ap*CPS, *Os*KO and *Sr*KS genes in CW1004.

The CPP could be obtained by catalyzing GGPP with Class II diterpene cyclases CPS, and CPP had three configurations as *syn* and *normal*/*ent* [29]. The reported CPS has the characteristics of low catalytic activity and non-specific catalytic products. In order to screen out an optimal CPS, all CPS functionally annotated as *ent*-copalyl diphosphate synthase were cloned from *A. paniculata* based on full-length transcriptome sequencing. A total of five full-length genes were found and contained conserved domains of DXDD (Fig. S4a), which were named *Ap*CPS1 to 5. The *Ap*CPS1-3 was previously described as *ent*-CPP synthetase [30] and *Ap*CPS4 and 5 were newly identified in this study. These five *Ap*CPS were correctly expressed in *E. coli* BL21 (Fig. S4b), and the results of phylogenetic tree analysis showed that they were very similar to those that had been reported to produce *ent*-CPP. The expression yield and distribution of *Ap*CPS1-5 were consistent with those of andrographolide (Fig. S4c), and they appeared to have the ability to produce *ent*-CPP (Fig. 4a, Table S3).

In order to verify the stereoconfiguration and catalytic ability of *Ap*CPS1-5 product *ent*-CPP, *Os*KS and *Sr*KO [9], which were specifically reacted with *ent*-CPP as substrate to generate *ent*-kaurenoic acid, were introduced into the downstream module of the strain CW1004. Compared with the *ent*-kaurenoic acid standard, it was found that *Ap*CPS1,2 and 4 could produce *ent*-kaurenoic acid (Fig. S5a). Compared with *Ap*CPS1 and 4, *Ap*CPS2 produced a maximum titer of 57.5 mg/L *ent*-kaurenoic acid (Fig. 4b). The *Ta*KSL1 can react with *normal*-CPP to produce iso-pimara-7,15-diene, with *syn*-CPP to produce *syn*-iso-pimara-7,15-diene, but not with *ent*-CPP [31]. In order to determine the catalytic specificity of *Ap*CPS2, *Ta*KSL1 was introduced into the downstream module of the strain CW1004 [31] and compared to the control group, with no new characteristic peaks generated (Fig. S5b), consistent with the previously reported *Ap*CPS2 specificity for the production of *ent*-CPP [32].

### 3.4 Rational design of *Ap*CPS2 and identification of *ent*-copalol

**Fig. 5:**
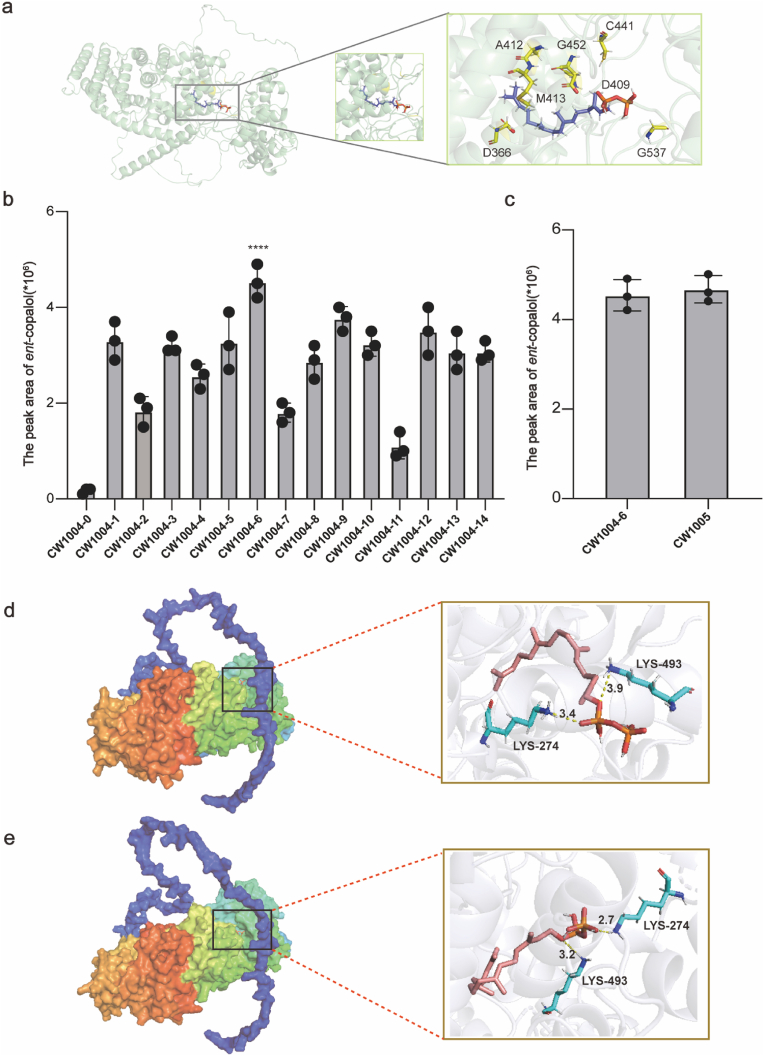
Effect of saturation mutation of *Ap*CPS2 on peak area of *ent*-copalol. (**a**) Design of saturated mutation sites for *Ap*CPS2. (**b**) Peak area of *ent*-copalol after fermentation at 13 mutation sites. (**c**) *Ap*CPS2^Met413Ser^ was integrated into the genome, and the peak area of *ent*-copalol was consistent with plasmid expression. **(d)** Residues in *Ap*CPS2 responsible for the formation of hydrogen bond with GGPP. (e) Residues in *Ap*CPS2^Met413Ser^ responsible for the formation of hydrogen bond with GGPP.

The *Ap*CPS2 was assembled with the medium-strong promoter GAL7 in pRS423 vector to form plasmid, which was transformed to CW1004, resulting into strain CW1004-1. After 96 h of fermentation and extraction, the culture broth of strain CW1004-1 showed the characteristic peak of *ent*-copalol compared with the control group CW1004-0 (Fig. S6a), but it could not be collected and qualitatively detected due to the low yield of *ent*-copalol (Fig. S6b). *ent*-copalol was not detected in the intracellular of CW1004-1. Further saturation mutations were designed, and after the successful docking of *Ap*CPS2 and GGPP at the β folding and α helix positions (Table S5). 13 mutants (Fig. 5a) at seven mutation sites with good virtual calculation results were screened and these 13 mutants were constructed on the pRS423 vector and transformed into the CW1004, resulting in strains CW1004-2 to CW1004-14. The peak area of substance 1 detected by CW1004-6 fermentation increased to 2.7*10^6^, so *Ap*CPS2^Met413Ser^ improved the catalytic ability of *Ap*CPS2 (Fig. 5b). *Ap*CPS2^Met413Ser^ was integrated into the genome to form CW1005, and the peak area was stable after fermentation (Fig. 5c). In order to explore the conformational change between *Ap*CPS2-GGPP and *Ap*CPS2^Met413Ser^-GGPP, the docking model before and after the mutation was obtained. The results showed that *Ap*CPS2^Met413Ser^ reshaped the hydrophobic pocket with more space to accommodate the matrix GGPP. The distance between the catalytic site of the phosphate group of the receptor GGPP and the key amino acid Lys-274 ranged from 3.4 to 2.7 Å, and the distance between the catalytic site of the phosphate group of the receptor GGPP and the key amino acid Lys-274 ranged from 3.9 to 3.2 Å (Fig. 5d and e). This remodeled conformation can reduce the catalytic barrier of *Ap*CPS2 to GGPP, thereby increasing its catalytic activity.

After the purification of CW1005 culture broth, it was found that pure compound 1 was a colorless, oily liquid. ESI-NS(*m/z*): [M+Na]^+^ calcd for C_20_H_34_ONa^+^ 313.2502 (Fig. S7), found 313.2504. The results of ^1^H NMR and ^13^C NMR were consistent with the *ent*-copalol reported in the literature [22](Figs. S8 and S9), compound 1 was described as *ent*-copalol. Further, 3.6 mg of *ent*-copalol was obtained, diluted with n-hexane and formulated into different concentration gradients for the preparation of standard curves and the detection of later yields.

### 3.5 Systematic optimization of *ent*-copalol biosynthesis

**Fig. 6:**
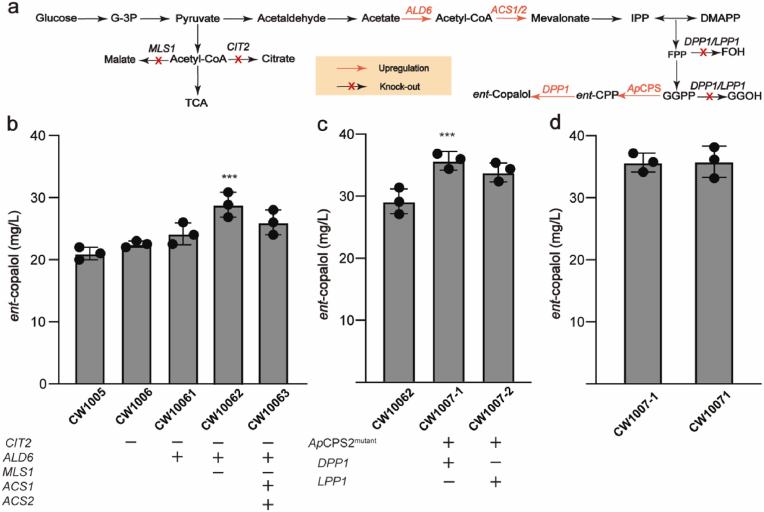
Effect of engineering acetyl-CoA metabolism and fusion-expression strategies on titer of *ent*-copalol. (**a**) Diagram of acetyl-CoA pathway and fusion expression in modified strains. (**b**) The ability of strains to modify different genes of acetyl-CoA to produce *ent*-copalol. (**c**) The titer of *ent*-copalol was compared after the fusion expression of *DPP1/LPP1* and *Ap*CPS2^Met413Ser^. (**d**) The titer of *ent*-copalol after fusion expression of *DPP1* and *Ap*CPS2^Met413Ser^ integrated into the genome.

In *S. cerevisiae*, the biosynthesis of terpenoids can be promoted not only by enhancing the MVA pathway, but also by enhancing acetyl-CoA. To explore the effect of regulating acetyl-CoA flux on *ent*-copalol synthesis, citrate synthase *CIT2* was knocked out and the endogenous acetaldehyde dehydrogenase *ALD6* was overexpressed to reduce the carbon loss of the precursor acetyl-CoA and the results showed in Fig. 6a that the titer of *ent*-copalol increased. The malate synthase *MLS1* was then knocked out to reduce the carbon loss of the precursor acetyl-CoA, resulting in CW10062, which could produce 29.0 mg/L *ent*-copalol, 37 % higher than that of the control group CW1005. The overexpressed acetyl-CoA synthases *ACS1* and *ACS2* were transformed into the CW10062, resulting in CW10063, with the titer of *ent-*copalol is lower in CW10063 than in CW10062 (Fig. 6b). The appropriate enhancement of acetyl-CoA can therefore show the advantages of *S. cerevisiae* in synthesizing terpenoids.

In *S. cerevisiae*, *DPP1* and *LPP1* were mainly responsible for the hydrolysis of isoprene-pyrophosphate phosphoryl [33], capable of converting FPP to farnesol and GGPP to GGOH [34]. The final step in the synthesis of *ent*-copalol in *S*. *cerevisiae* is *DPP1*/*LPP1* catalyzed *ent*-CPP formation. In order to reduce the loss of FPP and GGPP, the *LPP1* and *DPP1* orthotope genes were knocked out at the same time and fused with a short peptide linker with *Ap*CPS2^Met413Ser^ respectively (GGGGS*3) to form strain CW1007-1 (*Ap*CPS2^Met413Ser^-GGGGS*3-*DPP1*) and CW1007-2 (*Ap*CPS2^Met413Ser^-GGGGS*3-*LPP1*). After 96 h of fermentation, CW1007-1 produced 36.2 mg/L *ent*-copalol (Fig. 6c). The *Ap*CPS2^Met413Ser^-GGGGS*3-*DPP1* was integrated on the genome to form CW10071 (Fig. 6d). The results showed that the CW10071 produced 35.6 mg/L *ent*-copalol, which was the highest titer ever reported and indicated that the dephosphorylation ability of *DPP1* was more conducive to the formation of *ent*-copalol.

## 4 Discussion

Andrographolide, which is abundant in *A. paniculata*, has antipyretic, anti-inflammatory and antiviral effects [35]. It is currently mainly produced by the extraction from plants [36] and biosynthesis of andrographolide by microbial fermentation would enable more sustainable production. The understanding of andrographolide biosynthesis is however very limited and the downstream modification genes of *ent*-CPP and enzymes activity of CPS still need to be explored [37]. In this study, with the help of the excellent MVA precursor generation ability and endogenous dephosphatases of *S. cerevisiae* [33,34], a basic strain for the synthesis of diterpenes was constructed. The *Ap*CPS derived from *A. paniculata* was screened and *Ap*CPS was rationally modified. The culture broth was separated and purified and the configuration of *ent*-copalol was determined by HMR. The supply of acetyl-CoA and fusion-expression of *DPP1* and *Ap*CPS2^Met413Ser^ were further optimized to increase the titer of the *ent*-copalol. A strain that could *de novo* synthesize *ent*-copalol which is the unique precursor of andrographolide was constructed. Quantitative determination and the highest titer of *ent*-copalol were obtained in the CW10071 culture broth.

With the development of high-throughput sequencing technologies, RNASeq is now widely used in studying the metabolism of herbal medicines [38] and is a high-throughput approach for simultaneous identification of large numbers of genes [39], as well as being an effective method of identifying the biological functions of new genes during the biosynthesis of some diterpene products [26,38]. The transcriptome sequencing organization and number of transcripts in this study showed a higher number of transcripts compared to the leaf root transcriptome of *A. paniculata* previously reported [40]. Andrographolide biosynthesis was mainly influenced by GGPPs and *Ap*CPS [7,41]. Previous transcriptome data indicated the existence of at least four GGPPs genes in *A. paniculata* and only *Ap*GGPPs2 was involved in the biosynthesis of andrographolides [7]. The three *Ap*GGPPs screened in this study were all involved in the biosynthesis of GGPP in *S. cerevisiae* and had obvious catalytic activity, which may be caused by host differences.

In *A. paniculata*, the diterpene precursor GGPP is generated by GGPP synthase [8], GGPP is then catalyzed by Class II diterpene cyclase *Ap*CPS to form the parent ring structure *ent*-CPP [9]. The resulting parent nucleus is then catalyzed by Class I diterpenoid synthetases to form unique precursor structures, such as KS catalyzing *ent*-CPP to form *ent*-kaurene. But Class I diterpenoid synthetases involved in andrographolide synthesis have not yet been identified in *A. paniculata* [30]. The *Ap*CPS2 has been previously characterized to function as an *ent*-CPP synthase [32] and *Ap*CPS1 and *Ap*CPS3 have also been mined and qualified [30]. In addition to *Ap*CPS1 to 3, two full-length genes of *Ap*CPS4 and *Ap*CPS5 were screened in this study. Through the functional characterization of the introduction of strain CW1004 into the *ent*-kaurenoic acid pathway, it was found that *Ap*CPS1, 2 and 4 could produce *ent*-CPP. Through the introduction of *Ta*KSL enzyme in strain CW1004, it was concluded that *Ap*CPS2 and GGPP reaction generated only *ent*-configuration CPP.

Although *Ap*CPS2 has catalytic specificity, its catalytic activity is low [42]. Saturation mutations of designing enzymes are a strategy to improve enzyme activity [43]. In this study, the mutant *Ap*CPS2^Met413Ser^ screened in this study could significantly improve the titer of *ent*-copalol. Acetyl-CoA is an important chemical precursor [44], which can be used in the synthesis of isoprene [45]. The titer of diterpene could be increased by enhancing cytoplasmic acetyl-CoA in *S. cerevisiae* [46], this study found that excessive acetyl-CoA is unfavorable to the production of *ent*-copalol. In addition to the strategies used to increase the titer of *ent*-copalol, *Ap*CPS2 ^Met413Ser^ and *DPP1* were further fusion-expressed, which effectively reduced the loss of the *ent*-copalol. Therefore, appropriate strengthen acetyl-CoA and enhance catalytic specificity of *Ap*CPS in *S. cerevisiae* has referent significance for the biosynthesis of andrographolide in the future.

In summary, the genes related to *ent*-copalol biosynthesis, an important precursor of andrographolide, were extracted from the cDNA of *A. paniculata*. The *de novo* synthesis of *ent*-copalol in *S. cerevisiae* was realized by modifying the MVA pathway, screening *Ap*CPS and mutating *Ap*CPS2. The quantitative determination and configuration detection of *ent*-coaplol was carried out by means of isolation and purification. By moderately improving acetyl-CoA metabolic flow and fusion-expression, the yield of *ent*-copalol was further increased to 35.6 mg/L which currently is the only report of the *de novo* biosynthesis of *ent*-copalol and therefore the highest titer achieved. At present, there is reasonable speculation about the compounds in the andrographolide biosynthesis pathway, but the specific enzyme is still unknown [30]. Compared with other terpenoids, the titer of *ent*-copalol is lower, in addition to the low enzymatic activity of the CPS enzyme, it is also less region-selective to the substrate, and there are more by-products on the competitive pathway, which are the reasons for the low titer of *ent*-copalol. what is the future direction, the strategies such as enzyme truncation [47], remodeling of transmembrane structure [48], enhancement of cofactors and optimization of fermentation conditions will be used to increase titer. This study provides a reference for the heterologous identification of genes expressing synthetic andrographolide in *S. cerevisiae*, and also provides a platform for the analysis of andrographolide downstream genes.

## Authors’ contributions

Shan Li: Conceptualization, Methodology, Writing Original draft. Shuangshuang Luo: Validation. Xinran Yin: Performed homology modelling. Xingying Zhao: Validation. Xuyang Wang: Docking simulations. Song Gao: Validation. Sha Xu: Supervision, Funding acquisition. Jian Lu: coordination. Zhou Jingwen: Project administration, Writing – review & editing, Funding acquisition.

## Data availability

Data supporting the findings of this work are available within the manuscript and its Supplementary Information files.

## Declaration of competing interest

The authors declare that they have no known competing financial interests or personal relationships that could have appeared to influence the work reported in this paper.
