# Supplementary material for: Screening of ent-copalyl diphosphate synthase and metabolic engineering to achieve de novo biosynthesis of ent-copalol in Saccharomyces cerevisiae
Source: Synth Syst Biotechnol. 2024 Jun 18;9(4):784–92. doi: 10.1016/j.synbio.2024.06.005 (PMC11253141; doi:10.1016/j.synbio.2024.06.005)
Supplement: Multimedia component 1 [file mmc1.docx]

# Supplementary Tables

**Supplementary Table S1 Synthetic gene and screening genes.**

| **Name** | **Codon Optimized Sequence** |
| --- | --- |
| *Ap*GGPPs1 | MPTFLQNSFQFFLSQFHKTIPTMGSMNLVDTCAANSAAIFRPAPPILRSAFVGAARLPPCPIRLKKTSIAHSGGVAAILTEEDVKLAAGKEGRREFDFGRYVAEKAEFVNGALDGAVVMRQPAVIHEAMRYSLLAGGKRIRPMLCIAACEIVGGDPSAAVPAACAAEMIHTMSLIHDDLPCMDNDDLRRGKPTSHKMFGENVAVLAGDSLLAFAFEFIATGTKDVAPERIIACVDELAKAVGGDGLVAGQVADIKLTGNNADVGLDMLEFIHIHKTAVLLEASVVMGAILGGGSPQQVDKLRIFAQKIGLLFQVVDDILDVTMSSEELGKTAGKDLATDKTTYPKLLGVEGAREFANKLCEEAKEQLAEFDSDKAAPLAALAEYIGRRQN |
| *Ap*GGPPs2 | MSAVVNPIATWSRSISGGGFRPEQFNFLKPSRFRPMSISSAIIEETVATGKPHAFDLKKYMLNKASAVNAALEEAVPVRDPVTIHESMRYSLLAGGKRVRPMLCIAACELVGGEQAAALPAACAVEMIHTMSLMHDDLPCMDNDDLRRGKPTNHKVYGEDVAVLAGDALLAFAFEHLATATEDVPTNMVVSAIGELSRCIGAEGLVAGQVVDICSEGISEVGLELLEFIHLHKTAALLEGSVVLGAILGGATESEVERLRKFARCIGLLFQVVDDILDVTKCSEELGKTAGKDLVADKTTYPKLIGIEKSREFAEQLKREAKEQLEGFDPGKAAPLLALADYIASRDN |
| *Ap*GGPPs3 | MALLEGSVVLGAILGGATESEVERLRKFARCIGLLFQVVDDILDVTKCSEELGKTAGKDLVADKTTYPKLIGIEKSREFAEQLKREAKEQLEGFDPGKAAPLLALADYIASRDN |
| *Ap*CPS1 | MSLSLSGRSVFLVSAPHFPRPNMPLFSLLGSSPSPPPQLYIPAASPFPRTSGLFSVWRNTTNTGYPLLQCGAVSRAPAEGEYIDVILSGFPVRKWPSNIATEGDTQKDKYKGEEVRSNERRDRMVEMIKSMLRSMDDGEISISPYDTAWVALVEDIGGKGQPQFPTSIEWISNNQLEDGSWGDSAAYSAHDRILNTLACVVALRSWKMHQDKTDKGVTFIRKNIHRLDEEKEEHMPIGFEVALPSLIETAKKLGIDVPNDSPALQKIYARRELKLTRIPRDIMHKVPTTLLHSLEGMSSGLEWQELLKLQCPDGSFLFSPSSTAFALQQTKDENCLKYLLKHVRKFNGGVPNVYPVDLFERLWAVDRLQRLGISRYFQPEIEECLGYVHRYWTDKGICWARNSQVQDVDDTAMGFRLLRLHGYRVSAGIYNSCNLEQNIVNLRIYAYSTQSSAKIPAFIQSLYNASCKNFLCTTDVFKNFKQDEEFFCFAGQSNQAVTGMYNLYRASQVMFPGEVILAEARKFSHKFLQEKRANNELLDKWIITKDLPGEEFQVGFALDIPWYASLPRIETRFFLEQYGGGDDVWIGKTLYRMPYIDNRAYLDLAKLDYNNCQALHQSEWKSFQKWYRSCRLEEFGLSETTLVQTYYIAAASIFEPERLYERLAWAKTAILMETVMRHSDQKKLSKQQKHALVNEFKHCALLGERYKTRNNLIGTLVRSVNELSLDAPLARYADIQSHLHRAWQKWLSSWEEGDTGEGDAELLVCTLNLCGGGRSSHSRWSDKLLLSYPPYQPLVQIASRVCHHLRLSPTRKEEARMCEQKLQSGVSGGDIEGSMQELVRLALAKSHSELEFRVKQNFLLVARSYYYTAYCNPATINLHIAKVLFETVVLECCDDHTRRYF |
| *Ap*CPS2 | MSLSLSGRSVFLVSAPHFPRPNMPLFSLLGSSPSPPPQLYIPAASPFPRTSGLFSVWRNTTNTGYPLLQCGAVSRAPAEGEYIDVILSGFPVRKWPSNIATEGDTQKDKYKGEEVRSNERRDRMVEMIKSMLRSMDDGEISISPYDTAWVALVEDIGGKGQPQFPTSIEWISNNQLEDGSWGDSAAYSAHDRILNTLACVVALRSWKMHQDKTDKGVTFIRKNIHRLDEEKEEHMPIGFEVALPSLIETAKKLGIDVPNDSPALQKIYARRELKLTRIPRDIMHKVPTTLLHSLEGMSSGLEWQELLKLQCPDGSFLFSPSSTAFALQQTKDENCLKYLLKHVRKFNGGVPNVYPVDLFERLWAVDRLQRLGISRYFQPEIEECLGYVHRYWTDKGICWARNSQVQDVDDTAMGFRLLRLHGYRVSADVFKNFKQDEEFFCFAGQSNQAVTGMYNLYRASQVMFPGEVILAEARKFSHKFLQEKRANNELLDKWIITKDLPGEVGFALDIPWYASLPRIETRFFLEQYGGGDDVWIGKTLYRMPYIDNRAYLDLAKLDYNNCQALHQSEWKSFQKWYRSCRLEEFGLSETTLVQTYYIAAASIFEPERLYERLAWAKTAILMETVMRHSDQKKLSKQQKHALVNEFKHCALLGERYKTRNNLIGTLVRSVNELSLDAPLARYADIQSHLHRAWQKWLSSWEEGDTGEGDAELLVCTLNLCGGGRSSHSRWSDKLLLSYPPYQPLVQIASRVCHHLRLSPTRKEEARMCEQKLQSGVSGGDIEGSMQELVRLALAKSHSELEFRVKQNFLLVARSYYYTAYCNPATINLHIAKVLFETVVLECCDDHTRRYF |
| *Ap*CPS3 | MSLSLSGRSVFLVSAPHFPRPNMPLFSLLGSSPSPPPQLYIPAASPFPRTSVVAGLFSVWRNTTNTGYPLLQCGAVSRAPAEGEYIDVILSGFPVRKWPSNIATEGDTQKDKYKGEEVRSNERRDRMVEMIKSMLRSMDDGEISISPYDTAWVALVEDIGGKGQPQFPTSIEWISNNQLEDGSWGDSAAYSAHDRILNTLACVVALRSWKMHQDKTDKGVTFIRKNIHRLDEEKEEHMPIGFEVALPSLIETAKKLGIDVPNDSPALQKIYARRELKLTRIPRDIMHKVPTTLLHSLEGMSSGLEWQELLKLQCPDGSFLFSPSSTAFALQQTKDENCLKYLLKHVRKFNGGVPNVYPVDLFERLWAVDRLQRLGISRYWTDKGICWARNSQVQDVDDTAMGFRLLRLHGYRVSADVFKNFKQDEEFFCFAGQSNQAVTGMYNLYRASQVMFPGEVILAEARKFSHKFLQEKRANNELLDKWIITKDLPGEVGFALDIPWYASLPRIETRFFLEQYGGGDDVWIGKTLYRMPYIDNRAYLDLAKLDYNNCQALHQSEWKSFQKWYRSCRLEEFGLSETTLVQTYYIAAASIFEPERLYERLAWAKTAILMETVMRHSDQKKLSKQQKHALVNEFKHCALLGERCSKIGRYKTRNNLIGTLVRSVNELSLDAPLARYADIQSHLHRAWQKWLSSWEEGDTGEGDAELLVCTLNLCGGGRSSHSRWSDKLLLSYPPYQPLVQIASRVCHHLRLSPTRKEEARMCEQKLQSGVSGGDIEGSMQELVRLALAKSHSELEFRVKQNFLLVARSYYYTAYCNPATINLHIAKVLFETVVLECCDDHTRRYF |
| *Ap*CPS4 | MHKVPTTLLHSLEGMSSGLEWQELLKLQCPDGSFLFSPSSTAFALQQTKDENCLKYLLKHVRKFNGGVPNVYPVDLFERLWAVDRLQRLGISRYFQPEIEECLGYVHRYWTDKGICWARNSQVQDVDDTAMGFRLLRLHGYRVSADVFKNFKQDEEFFCFAGQSNQAVTGMYNLYRASQVMFPGEVILAEARKFSHKFLQEKRANNELLDKWIITKDLPGEVGFALDIPWYASLPRIETRFFLEQYGGGDDVWIGKTLYRMPYIDNRAYLDLAKLDYNNCQALHQSEWKSFQKWYRSCRLEEFGLSETTLVQTYYIAAASIFEPERLYERLAWAKTAILMETVMRHSDQKKLSKQQKHALVNEFKHCALLGERYKTRNNLIGTLVRSVNELSLDAPLARYADIQSHLHRAWQKWLSSWEEGDTGEGDAELLVCTLNLCGGGRSSHSRWSDKLLLSYPPYQPLVQIASRVCHHLRLSPTRKEEARMCEQKLQSGVSGGDIEGSMQELVRLALAKSHSELEFRVKQNFLLVARSYYYTAYCNPATINLHIAKVLFETVVLECCDDHTRRYF |
| *Ap*CPS5 | MGFRLLRLHGYRVSADVFKNFKQDEEFFCFAGQSNQAVTGMYNLYRASQVMFPGEVILAEARKFSHKFLQEKRANNELLDKWIITKDLPGEVGFALDIPWYASLPRIETRFFLEQYGGGDDVWIGKTLYRMPYIDNRAYLDLAKLDYNNCQALHQSEWKSFQKWYRSCRLEEFGLSETTLVQTYYIAAASIFEPERLYERLAWAKTAILMETVMRHSDQKKLSKQQKHALVNEFKHCALLGERCSKIGRYKTRNNLIGTLVRSVNELSLDAPLARYADIQSHLHRAWQKWLSSWEEGDTGEGDAELLVCTLNLCGGGRSSHSRWSDKLLLSYPPYQPLVQIASRVCHHLRLSPTRKEEARMCEQKLQSGVSGGDIEGSMQELVRLALAKSHSELEFRVKQNFLLVARSYYYTAYCNPATINLHIAKVLFETVVLECCDDHTRRYF |

**Supplementary Table S2 Strains and plasmids used in this study.**

| **Strain** | **Host strain** | **Description** | **Source** |
| --- | --- | --- | --- |
| C800 | CEN.PK2-1D | Δ*gal80*::G418 | Preserved in this laboratory |
| CW1000 | C800 | Δ*adh1*::TEF1p-EGR20^F96c^-CYC1t | This study |
| CW1001 | CW1000 | *Ty4*::SHM2p- tHMG1-TERR22t-IDI1-TDH3p | This study |
| CW10011 | CW1001 | *Δerg9p*::HXT1p-ERG9 -CYC1t |  |
| CW1002 | CW1001 | *Δgpd1* | This study |
| CW1003 | CW1002 | *Δgpd2* | This study |
| CW1003-0 | CW1002 | pRS423 | This study |
| CW1003-1 | CW1002 | pRS423--P_GAL7_-*Tm*GGPPs- TEF1t | This study |
| CW1003-2 | CW1002 | pRS423--P_GAL7_-*Pa*GGPPs- TEF1t | This study |
| CW1003-3 | CW1002 | pRS423--P_GAL7_-*Ap*GGPPs1- TEF1t | This study |
| CW1003-4 | CW1002 | pRS423--P_GAL7_-*Ap*GGPPs2- TEF1t | This study |
| CW1003-5 | CW1002 | pRS423--P_GAL7_-*Ap*GGPPs3- TEF1t | This study |
| CW1004 | CW1003 | *Δypl062w*::GAL7p-*Pa*GGPPs -TEF1t | This study |
| CW1004-0 | CW1004 | pRS423 | This study |
| CW1004-1 | CW1004 | pRS423-P_GAL7_-*Ap*CPS2- CYC1t | This study |
| CW1004-2 | CW1004 | pRS423-P_GAL7_-*Ap*CPS2^ASP366MET^- CYC1t | This study |
| CW1004-3 | CW1004 | pRS423-P_GAL7_-*Ap*CPS2^GYL452TRP^- CYC1t | This study |
| CW1004-4 | CW1004 | pRS423-P_GAL7_-*Ap*CPS2^MET413LEU^- CYC1t | This study |
| CW1004-5 | CW1004 | pRS423-P_GAL7_-*Ap*CPS2^ASP3409ILE^- CYC1t | This study |
| CW1004-6 | CW1004 | pRS423-P_GAL7_-*Ap*CPS2^MET413SER^- CYC1t | This study |
| CW1004-7 | CW1004 | pRS423-P_GAL7_-*Ap*CPS2^ASP366ASN^- CYC1t | This study |
| CW1004-8 | CW1004 | pRS423-P_GAL7_-*Ap*CPS2^GLY537THR^- CYC1t | This study |
| CW1004-9 | CW1004 | pRS423-P_GAL7_-*Ap*CPS2^ALA412TYR^- CYC1t | This study |
| CW1004-10 | CW1004 | pRS423-P_GAL7_-*Ap*CPS2^MET413ASN^- CYC1t | This study |
| CW1004-11 | CW1004 | pRS423-P_GAL7_-*Ap*CPS2^CYS441AGR^- CYC1t | This study |
| CW1004-12 | CW1004 | pRS423-P_GAL7_-*Ap*CPS2^GLY537GLN^- CYC1t | This study |
| CW1004-13 | CW1004 | pRS423-P_GAL7_-*Ap*CPS2^ASP336THR^- CYC1t | This study |
| CW1004-14 | CW1004 | pRS423-P_GAL7_-*Ap*CPS2^GLY452PHE^- CYC1t | This study |
| CW1005 | CW1004 | *Δyjl064::* GAL7Pp-*Ap*CPS2^MET413SER^- CYC1t | This study |
| CW1006 | CW1005 | *Δcit2* | This study |
| CW10061 | CW1005 | *Δcit2::* TEF1p-ALD6 – CYC1t | This study |
| CW10062 | CW10061 | *Δmls1* |  |
| CW10063 | CW10061 | *Δmls1::* TER22-ACS1-GAL1.10p-ACS2 - CYC1t |  |
| CW1007 | CW10062 | Δ*lpp1*; *Δdpp1* | This study |
| CW1007-1 | CW1007 | pRS423-P_GAL7_-*Ap*CPS2 ^MET413SER^ -DPP1-TEF1t | This study |
| CW1007-2 | CW1007 | pRS423-P_GAL7_-*Ap*CPS2 ^MET413SER^ -LPP1-TEF1t | This study |
| CW10071 | CW10062 | Δ*Apcps*2^MET413SER^::GAL7p-*Ap*CPS2 ^MET413SER^ -DPP1-TEF1t | This study |
| pET28a-1 | BL21 | pET28a-P_T7_-*Ap*CPS1-6*His-T7t | This study |
| pET28a-2 | BL21 | pET28a-P_T7_-*Ap*CPS2-6*His-T7t | This study |
| pET28a-3 | BL21 | pET28a-P_T7_-*Ap*CPS3-6*His-T7t | This study |
| pET28a-4 | BL21 | pET28a-P_T7_-*Ap*CPS4-6*His-T7t | This study |
| pET28a-5 | BL21 | pET28a-P_T7_-*Ap*CPS5-6*His-T7t | This study |
| pRS423-1 | CW1004 | pRS423-P_GAL7_-*Ap*CPS1-*OS*KS-GAL1.10p-*Sr*KO-TEF1t | This study |
| pRS423-2 | CW1004 | pRS423-P_GAL7_-*Ap*CPS2-*OS*KS-GAL1.10p-*Sr*KO-TEF1t | This study |
| pRS423-3 | CW1004 | pRS423-P_GAL7_-*Ap*CPS3-*OS*KS-GAL1.10p-*Sr*KO-TEF1t | This study |
| pRS423-4 | CW1004 | pRS423-P_GAL7_-*Ap*CPS4-*OS*KS-GAL1.10p-*Sr*KO-TEF1t | This study |
| pRS423-5 | CW1004 | pRS423-P_GAL7_-*Ap*CPS5-*OS*KS-GAL1.10p-*Sr*KO-TEF1t | This study |
| pRS423-6 | CW1004 | pRS423- P_GAL7_-*Ap*CPS2-TEF1t | This study |
| pRS423-7 | CW1004 | P_GAL7_-*Ap*CPS2 -GAL1.10p-*TaKSL1*-TEF1t | This study |

**Supplementary Table S3 Primers used in this study.**

| **Name** | **Sequence (5ʹ-3ʹ) ^*^** |
| --- | --- |
| CW1000-CYC1t-F | gtttacaagagaagtaaataatcatgtaattagttatgtcacgcttacattcacg |
| CW1000-CYC1t-R | gcttttggttcaatttttgtgcaaattaaagccttcgagcgtcc |
| CW1000-DOWN-F | aaggctttaatttgcacaaaaattgaaccaaaagctatagtagtcatatatatatatat |
| CW1000-DOWN-R | ctatgaccatgattacgccaaggaagtggaatgatatagatcagatagctacatcgc |
| CW1000-ERG20/96-F | aagcatagcaatctaatctaagatggcttcagaaaaagaaattaggagagagag |
| CW1000-ERG20/96-R | tgacataactaattacatgattatttacttctcttgtaaaccttgttcaaaaacgc |
| CW1000-TEF1p-F | atttaataagaacagatcatcatagcttcaaaatgtttctactccttttttactcttc |
| CW1000-TEF1p-R | atttctttttctgaagccatcttagattagattgctatgctttctttctaatgagcaa |
| CW1000-UP-F | gccagtgaattagaactcggcgaaccagattgtcaaataaacattgttaattgatga |
| CW1000-UP-R | aacattttgaagctatgatgatctgttcttattaaattcaaacaatttaggtacgaaag |
| CW1000-cas9-F | acaatgaagatttttcgattgttttagagctagaaatagcaagttaaaataaggctagt |
| CW1000-cas9-R | aatcgaaaaatcttcattgtgatcatttatctttcactgcggagaagtttc |
| CW1001-IDI1-F | Accttgaggcttttgacattttatagcattctatgaatttgcctgtcattttccac |
| CW1001-IDI1-R | aacacacataaacaaacaaaatgactgccgacaacaatagtatgcc |
| CW1001-PDH3p-F | ctattgttgtcggcagtcattttgtttgtttatgtgtgtttattcgaaactaagttctt |
| CW1001-PDH3p-R | ccttttcggttagagcggatataaaaaacacgctttttcagttcgagtttatca |
| CW1001-SHM2p-F | acgtatctaccaacggaatgcgtcttggcaggcaatagtgtac |
| CW1001-SHM2p-R | accaattggtctgcagccatatcaacttatgatgagcgtcggatagagt |
| CW1001-TER22t-F | tcacctgcattaaatcctaaataggttggcttccatgttggct |
| CW1001-TER22t-R | aaattcatagaatgctataaaatgtcaaaagcctcaaggtgcc |
| CW1001-tHMG1-F | gacgctcatcataagttgatatggctgcagaccaattggtg |
| CW1001-tHMG1-R | ccaacatggaagccaacctatttaggatttaatgcaggtgacggacc |
| CW10011-DOWN-F | ctagttgatatacgtaaaatcatgggaaagctattacaattggcattgc |
| CW10011-DOWN-R | gcaacccattcaagttgtaattttcatccaag |
| CW10011-HXT1p-F | taattacatgactcgagcgaagcggcatggcagg |
| CW10011-HXT1p-R | agctttcccatgattttacgtatatcaactagttgacgattatgatatctttatagatt |
| CW10011-TEFt-F | gctctgactcagtacatttcatagggtaccggccgcaaattaaagc |
| CW10011-TEFt-R | cagtccctgccatgccgcttcgctcgagtcatgtaattagttatgtcacgc |
| CW10011-UP-F | gccagtgaattagaactcggcacaatgtagggctatatatgctggagc |
| CW10011-UP-R | tcgaaggctttaatttgcggccggtaccctatgaaatgtactgagtcagagcacgc |
| CW1001-cas9-F | tcggtattgttgttgaagatgttttagagctagaaatagcaagttaaaataaggctag |
| CW1001-cas9-R | atcttcaacaacaataccgagatcatttatctttcactgcggagaagtttc |
| CW1002-DOWN-F | gtgtccaattgctatcctcttagttgtggtttcgtcgaaggtctagg |
| CW1002-DOWN-R | ctaatcttcatgtagatctaattcttcaatcatgtccg |
| CW1002-UP-F | gccagtgaattagaactcggatgtctgctgctgctgatagattaaactt |
| CW1002-UP-R | agaccttcgacgaaaccacaactaagaggatagcaattggacacctttagca |
| CW1002-cas9-F | gattcacacgtcagagctatttttagagctagaaatagcaagttaaaataaggctag |
| CW1002-cas9-R | atagctctgacgtgtgaatccatttatctttcactgcggagaagtttc |
| CW1003-CYC1t-F | agctgtcagaattaagttgtaatcatgtaattagttatgtcacgcttacattcacg |
| CW1003-CYC1t-R | gagggggggggagagtgtgcaaattaaagccttcgagcgtcc |
| CW1003-DOWN-F | aaggctttaatttgcacactctccccccccc |
| CW1003-DOWN-R | Tatgaccatgattacgccaagcctgcctttaaatgactcttccatacgg |
| CW1003-UP-F | gccagtgaattagaactcggcgccttagcctctagccatagc |
| CW1003-UP-R | gtagaaacattttgaagctatgtgataaggaaggggagcgaaggaaaag |
| CW1003-cas9-F | gtgtctaaaagggttcgagtgttttagagctagaaatagcaagttaaaataaggctagt |
| CW1003-cas9-F | actcgaaccctttagacacgatcatttatctttcactgcggagaagtttc |
| CW003-0-F | actcactatagggcgaattgggtaccggccgcaaattaaagc |
| CW003-0-R | gctttaatttgcggccggtacccaattcgccctatagtgagtcgtattacg |
| CW1003-1-GAL7p-F | gactcactatagggcgaattgtttgccagcttactatccttcttgaaaatatgc |
| CW1003-1-GAL7p-R | gctttcaggcaaaactaattttgagggaatattcaactgtttttttttatcatgttgat |
| CW1003-1-TEFt-F | gccattgcggtataagccatggtaccggccgcaaattaaagc |
| CW1003-1-TEFt-R | actaaagggaacaaaagctgctcgagtcatgtaattagttatgtcacgcttac |
| CW1003-1-TmGGPPs-F | agttgaatattccctcaaaattagttttgcctgaaagcgatgtaatcg |
| CW1003-1-TmGGPPs-R | ctttaatttgcggccggtaccatggcttataccgcaatggcag |
| CW1003-2-GAL7p-F | gactcactatagggcgaattgtttgccagcttactatccttcttgaaaatatgc |
| CW1003-2-TEFt-R | actaaagggaacaaaagctgctcgagtcatgtaattagttatgtcacgcttac |
| CW1003-2-GAL7p-R | cagcacaaactgtcattttgagggaatattcaactgtttttttttatcatgttgatg |
| CW1003-2-PaGGPPs-F | agttgaatattccctcaaaaatgacagtttgtgctgaacaacatgttaattttattc |
| CW1003-2-PaGGPPs-R | tttaatttgcggccggtacctta agaaacagcagctaatttcttttcaaaccaagc |
| CW1003-2-TEFt-F | aattagctgctgtttcttaaggtaccggccgcaaattaaagc |
| CW1003-3-GAL7p-F | gactcactatagggcgaattgtttgccagcttactatccttcttgaaaatatgc |
| CW1003-3-TEFt-R | actaaagggaacaaaagctgctcgagtcatgtaattagttatgtcacgcttac |
| CW1003-3-ApGGPP1-F | agttgaatattccctcaaaa ccgacgtttctccaaaattcatttcaattttttc |
| CW1003-3-ApGGPP1-R | ctttaatttgcggccggtacc tcaattctgcctccgaccaatgtac |
| CW1003-3-GAL7p-R | ttttggagaaacgtcggttttgagggaatattcaactgtttttttttatcatgttgat |
| CW1003-3-TEF1t-R | ttggtcggaggcagaattgaggtaccggccgcaaattaaagc |
| CW1003-4-GAL7p-F | gactcactatagggcgaattgtttgccagcttactatccttcttgaaaatatgc |
| CW1003-4-TEFt-R | actaaagggaacaaaagctgctcgagtcatgtaattagttatgtcacgcttac |
| CW1003-4-ApGGPP2-F | agttgaatattccctcaaaaatgagtgcggtggtgaatcc |
| CW1003-4-ApGGPP2-R | tttaatttgcggccggtacctcaattatccctagaagcaatgtaatcagccaag |
| CW1003-4-GAL7p-R | tcaccaccgcactcatttttgagggaatattcaactgtttttttttatcatgttgatg |
| CW1003-4-TEFt-F | ttgcttctagggataattgaggtaccggccgcaaattaaagc |
| CW1003-5-ApGGPP3-F | agttgaatattccctcaaaaaacctactcctagattcaaatccaattcaacaagt |
| CW1003-5-ApGGPP3-R | tttaatttgcggccggtacctcaattatccctagaagcaatgtaatcagccaag |
| CW1003-5-GAL7p-F | gactcactatagggcgaattgtttgccagcttactatccttcttgaaaatatgc |
| CW1003-5-GAL7p-R | gaatctaggagtaggttttttgagggaatattcaactgtttttttttatcatgttgatg |
| CW1003-5-TEFt-F | ttgcttctagggataattgaggtaccggccgcaaattaaagc |
| CW1003-5-TEFt-R | actaaagggaacaaaagctgctcgagtcatgtaattagttatgtcacgcttac |
| CW1004-DOWN-F | caccgaccatgtgggcaaattcgtaata |
| CW1004-DOWN-R | atgaccatgattacgccaagttcagtggaaggatcttcgacggg |
| CW1004-GAL7p-F | cccctcacgtaagggctttgccagcttactatccttcttgaaaatatgc |
| CW1004-GAL7p-R | cagcacaaactgtcatttttgagggaatattcaactgtttttttttatcatgttgatg |
| CW1004-PaGGPPs-F | agttgaatattccctcaaaaatgacagtttgtgctgaacaacatgttaattttattc |
| CW1004-PaGGPPs-R | tttaatttgcggccggtaccttaagaaacagcagctaatttcttttcaaaccaagc |
| CW1004-TEFt-F | aattagctgctgtttcttaaggtaccggccgcaaattaaagc |
| CW1004-TEFt-R | gtttacaagagaagtaaataatcatgtaattagttatgtcacgcttacattcacg |
| CW1004-UP-F | gttactgtcatgtgaataaaacttatgtatattgctaacttact |
| CW1004-UP-R | gaaggatagtaagctggcaaagcccttacgtgaggggca |
| CW1004-cas9-F | cccagaaagaaaaaagagggtgttttagagctagaaatagcaagttaaaataaggct |
| CW1004-cas9-R | accctcttttttctttctgggatcatttatctttcactgcggagaagtttcgaa |
| CW1004-1-APS2-F | agttgaatattccctcaaaagtctctctctccctcagtggtcg |
| CW1004-1-APS2-R | tttaatttgcggccggtacctcagaagtaacggcgggtatgg |
| CW1004-1-GAL7p-F | gactcactatagggcgaattgtttgccagcttactatccttcttgaaaatatgc |
| CW1004-1-GAL7p-R | ctgagggagagagagacttttgagggaatattcaactgtttttttttatcatgttgatg |
| CW1004-1-TEF1t-F | atacccgccgttacttctgaggtaccggccgcaaattaaagc |
| CW1004-1-TEF1t-R | actaaagggaacaaaagctgctcgagtcatgtaattagttatgtcacgcttac |
| CW1005-APS2/413-F | agttgaatattccctcaaaagtctctctctccctcagtggtcg |
| CW1005-APS2/413-R | tttaatttgcggccggtacctcagaagtaacggcgggtatgg |
| CW1005-DOWN-F | taactaattacatgactcgagtcacatcgagcgagctatcctg |
| CW1005-DOWN-R | atgaccatgattacgccaaggacgccaaccagctacctcataa |
| CW1005-GAL7p-F | gagtaaacttcattagggggctttgccagcttactatccttcttgaaaatatgc |
| CW1005-GAL7p-R | ctgagggagagagagacttttgagggaatattcaactgtttttttttatcatgttgatg |
| CW1005-TEF1t-F | atacccgccgttacttctgaggtaccggccgcaaattaaagc |
| CW1005-TEFt-R | tcaggatagctcgctcgatgtgactcgagtcatgtaattagttatgtcacgctt |
| CW1005-UP-F | gccagtgaattagaactcggcatcaatatcccttaactttggaaagaggttcc |
| CW1005-UP-R | tcaagaaggatagtaagctggcaaagccccctaatgaagtttactccgc |
| CW1005-cas9-F | tgctacagaattcttcgtccgttttagagctagaaatagcaagttaaaataaggctag |
| CW1005-cas9-R | ggacgaagaattctgtagcagatcatttatctttcactgcggagaagtttcg |
| CW1006-DOWN-F | aactaattacatgactcgagttggattacatcctacttttacacccctct |
| CW1006-DOWN-R | ccatgattacgccaagcgtaaaacaagtaaaaatgtaggatgttatagtatcaactag |
| CW1006-TEF1t-F | aataatactagtaacaagaaaaggtaccggccgcaaattaaagc |
| CW1006-TEF1t-R | aaaagtaggatgtaatccaactcgagtcatgtaattagttatgtcacgcttac |
| CW1006-UP-F | ggccagtgaattagaactcgggagttgttgccacaacataagccg |
| CW1006-UP-R | cggccggtaccttttcttgttactagtattattaaaacaaaaagttttgagaacctgtt |
| CW1006-cas9-F | tgtattcaaagatggcaaaagttttagagctagaaatagcaagttaaaataaggctag |
| CW1006-cas9-R | ttttgccatctttgaatacagatcatttatctttcactgcggagaagttt |
| CW1006-DOWN-R | ccatgattacgccaagcgtaaaacaagtaaaaatgtaggatgttatagtatcaactagc |
| CW10061-ALD6-F | aagcatagcaatctaatctaagatgactaagctacactttgacactgctg |
| CW10061-ALD6-R | cgtgacataactaattacatgattacaacttaattctgacagcttttacttcagtgta |
| CW10061-CYC1t-F | agctgtcagaattaagttgtaatcatgtaattagttatgtcacgcttacattcacg |
| CW10061-CYC1t-R | aaaagtaggatgtaatccaagcaaattaaagccttcgagcgtcc |
| CW10061-DOWN-F | cgctcgaaggctttaatttgcttggattacatcctacttttacacccctctg |
| CW10061-TEF1p-R | gtcaaagtgtagcttagtcatcttagattagattgctatgctttctttctaatgagca |
| CW10061-TEF1t-F | atactagtaacaagaaaacatagcttcaaaatgtttctactccttttttactcttcca |
| CW10061-UP-F | ggccagtgaattagaactcgggagttgttgccacaacataagccg |
| CW10061-UP-R | ttgaagctatgttttcttgttactagtattattaaaacaaaaagttttgagaacctgtt |
| CW10062-DOWN-F | taactaattacatgactcgagtctcccttgccccagtgtacac |
| CW10062-DOWN-R | tcacgacgttgtaaaacgaccgaactgatacccgacgccg |
| CW10062-TEFt-F | agcacataaaagaattaagaaaggtaccggccgcaaattaaagc |
| CW10062-TEFt-R | tgtacactggggcaagggagactcgagtcatgtaattagttatgtcacgcttac |
| CW10062-UP-F | agggaacaaaagctggagctcgctatcggctcaatggaaatcccc |
| CW10062-UP-R | gcggccggtacctttcttaattcttttatgtgcttttactactttgtttagttcaaaac |
| CW10062-cas9-F | agaaaatcgtacaaattgaagttttagagctagaaatagcaagttaaaataaggctag |
| CW10062-cas9-R | ttcaatttgtacgattttctgatcatttatctttcactgcggagaagtttc |
| CW10063-ACS1-F | accttgaggcttttgacattttacaacttgaccgaatcaattagatgtctaacaatgc |
| CW10063-ACS1-R | caaggagaaaaaactata atgtcgccctctgccgt |
| CW10063-ACS2-F | atttttgaaaattcaatataaatgacaatcaaggaacataaagtagtttatgaagctc |
| CW10063-ACS2-R | taatttgcggccggtaccttatttctttttttgagagaaaaattggttctctacagcag |
| CW10063-DOWN-F | taactaattacatgactcgagtctcccttgccccagtgtacac |
| CW10063-DOWN-R | tcacgacgttgtaaaacgaccgaactgatacccgacgccg |
| CW10063-GAL.10p-F | cagagggcgacattatagttttttctccttgacgttaaagtatagaggtatattaacaa |
| CW10063-GAL.10p-R | ccttgattgtcatttatattgaattttcaaaaattcttactttttttttggatggacgc |
| CW10063-TEFt-F | tctctcaaaaaaagaaataaggtaccggccgcaaattaaagc |
| CW10063-TEFt-R | tgtacactggggcaagggagactcgagtcatgtaattagttatgtcacgcttac |
| CW10063-TER22t-F | gcacataaaagaattaagaaaataggttggcttccatgttggctat |
| CW10063-TER22t-R | ttgattcggtcaagttgtaaaatgtcaaaagcctcaaggtgcca |
| CW10063-UP-F | agggaacaaaagctggagctcgctatcggctcaatggaaatcccc |
| CW10063-UP-R | aagccaacctattttcttaattcttttatgtgcttttactactttgtttagttcaaaac |
| CW1007-DOWNlpp1-F | gctcgaaggctttaatttgccaagcggacattcaagtttcatagtcagta |
| CW1007-DOWNlpp1-R | atgaccatgattacgccaagctaaacactaaccggtgaaggaagtatgtct |
| CW1007-lpp1-CYC1t-F | tcacttcatgcatactagcatcatgtaattagttatgtcacgcttacattcacg |
| CW1007-lpp1-CYC1t-R | gaaacttgaatgtccgcttggcaaattaaagccttcgagcgtcc |
| CW1007-UPlpp1-F | gccagtgaattagaactcggtgatctctgtcatggcggatgagaaa |
| CW1007-UPlpp1-R | tgacataactaattacatgatgctagtatgcatgaagtgaaaatcgttc |
| CW1007-cas9-lpp1-F | gattataagcataaatgctggttttagagctagaaatagcaagttaaaataaggctagt |
| CW1007-cas9-lpp1-R | cagcatttatgcttataatcgatcatttatctttcactgcggagaagtttcgaac |
| CW1007-dpp1-CYC1t-F | aatcatagcaaacgaccaaatcatgtaattagttatgtcacgcttacattcacg |
| CW1007-dpp1-CYC1t-R | gtcatgtggagtatatattcttttttattcgcaaattaaagccttcgagcgtc |
| CW1007-DOWNdpp1-F | gctcgaaggctttaatttgcgaataaaaaagaatatatactccacatgacatacgaaat |
| CW1007-DOWNdpp1-R | atgaccatgattacgccaagttatacatagtatgtgttaaggggaacggaaaa |
| CW1007-UPdpp1-F | gccagtgaattagaactcggactattattaaggcgcttctgtttttagtc |
| CW1007-UPdpp1-R | tgacataactaattacatgatttggtcgtttgctatgatttaattctgattc |
| CW1007-cas9-dpp1-F | cgattcagatgtcaccctgggttttagagctagaaatagcaagttaaaataaggc |
| CW1007-cas9-dpp1-R | ccagggtgacatctgaatcggatcatttatctttcactgcggagaag |
| CW1007-1-GAL7p-F | gactcactatagggcgaattgtttgccagcttactatccttcttgaaaatatgc |
| CW1007-1-APS2/413-F | agttgaatattccctcaaaagtctctctctccctcagtggtcg |
| CW1007-1-APS2/413-R | taataaacgaaactctgttcatgaagtaacggcgggtatggtca |
| CW1007-1-DPP1-F | accatacccgccgttacttcatgaacagagtttcgtttattaaaacgccttt |
| CW1007-1-DPP1-R | ctttaatttgcggccggtaccttacataccttcatcggacaaaggatgtaatt |
| CW1007-1-GAL7p-R | ctgagggagagagagacttttgagggaatattcaactgtttttttttatcatgttgatg |
| CW1007-1-TEF1t-R | actaaagggaacaaaagctgctcgagtcatgtaattagttatgtcacgcttac |
| CW1007-1-TEFt-F | ttgtccgatgaaggtatgtaaggtaccggccgcaaattaaagc |
| CW1007-2-GAL7p-F | gactcactatagggcgaattgtttgccagcttactatccttcttgaaaatatgc |
| CW1007-2-ACS2/413-F | atccgccatgacagagatcatgaagtaacggcgggtatggtcat |
| CW1007-2-APS2/413-F | agttgaatattccctcaaaagtctctctctccctcagtggtcg |
| CW1007-2-GAL7p-R | ctgagggagagagagacttttgagggaatattcaactgtttttttttatcatgttgat |
| CW1007-2-LPP1-F | accatacccgccgttacttcatgatctctgtcatggcggatgag |
| CW1007-2-LPP1-R | ggctttaatttgcggccggtaccctaaacactaaccggtgaaggaagtatgt |
| CW1007-2-TEFt-F | ccttcaccggttagtgtttagggtaccggccgcaaattaaagc |
| CW1007-2-TEFt-R | actaaagggaacaaaagctgctcgagtcatgtaattagttatgtcacgcttac |
| CW10071-APS2/413-F | agttgaatattccctcaaaagtctctctctccctcagtggtcg |
| CW10071-APS2/413-R | taataaacgaaactctgttcatgaagtaacggcgggtatggtca |
| CW10071-DOWN-F | taactaattacatgactcgagtcacatcgagcgagctatcctg |
| CW10071-DOWN-R | atgaccatgattacgccaaggacgccaaccagctacctcataa |
| CW10071-DPP1-F | accatacccgccgttacttcatgaacagagtttcgtttattaaaacgccttt |
| CW10071-DPP1-R | ctttaatttgcggccggtaccttacataccttcatcggacaaaggatgtaatt |
| CW10071-GAL7p-F | gagtaaacttcattagggggctttgccagcttactatccttcttgaaaatatgc |
| CW10071-GAL7p-R | ctgagggagagagagacttttgagggaatattcaactgtttttttttatcatgttgat |
| CW10071-TEFt-F | ttgtccgatgaaggtatgtaaggtaccggccgcaaattaaagc |
| CW10071-TEFt-R | tcaggatagctcgctcgatgtgactcgagtcatgtaattagttatgtcacgctt |
| CW10071-UP-F | gccagtgaattagaactcggcatcaatatcccttaactttggaaagaggttcc |
| CW10071-UP-R | tcaagaaggatagtaagctggcaaagccccctaatgaagtttactccgc |
| CW10071-cas9-F | acattactgcacagcttagagttttagagctagaaatagcaagttaaaataaggc |
| CW10071-cas9-R | tctaagctgtgcagtaatgt gatcatttatctttcactgcggagaag |
| pET28a-1F | catacccgccgttacttccaccaccaccaccaccactgagatccggctgctaacaaagc |
| pET28a-1R | gggagagagagaccatggtatatctccttcttaaagttaaacaaaattatttctagagg |
| pET28a-ApCPS1-F | ctttaagaaggagatataccatggtctctctctccctcagtggtcg |
| pET28a-ApCPS1-R | tagcagccggatctcagtggtggtggtggtggtggaagtaacggcgggtatggtcatc |
| pET28a-2-F | catacccgccgttacttccaccaccaccaccaccactgagatccggctgctaacaaagc |
| pET28a-2-R | gcctgaatcccatcatggtatatctccttcttaaagttaaacaaaattatttctagag |
| pET28a-ApCPS2-F | ctttaagaaggagatataccatgatgggattcaggcttctgaggtt |
| pET28a-ApCPS2-R | tagcagccggatctcagtggtggtggtggtggtggaagtaacggcgggtatggtcatc |
| pET28a-3-F | atacccgccgttacttccaccaccaccaccaccactgagatccggctgctaacaaagc |
| pET28a-3-R | gggagagagagaccatggtatatctccttcttaaagttaaacaaaattatttctagagg |
| pET28a-ApCPS3-F | ctttaagaaggagatataccatggtctctctctccctcagtggtcg |
| pET28a-ApCPS3-R | tagcagccggatctcagtggtggtggtggtggtggaagtaacggcgggtatggtcatc |
| pET28a-4-F | catacccgccgttacttccaccaccaccaccaccactgagatccggctgctaacaaagc |
| pET28a-4-R | gcaccttgtgcatcatggtatatctccttcttaaagttaaacaaaattatttctagagg |
| pET28a-ApCPS4-F | ctttaagaaggagatataccatgatgcacaaggtgcccacaac |
| pET28a-ApCPS4-R | tagcagccggatctcagtggtggtggtggtggtggaagtaacggcgggtatggtcatc |
| pET28a-5-F | catacccgccgttacttccaccaccaccaccaccactgagatccggctgctaacaa |
| pET28a-5-R | gggagagagagaccatggtatatctccttcttaaagttaaacaaaattatttctagag |
| pET28a-ApCPS5-F | ctttaagaaggagatataccatggtctctctctccctcagtggtcg |
| pET28a-ApCPS5-R | tagcagccggatctcagtggtggtggtggtggtggaagtaacggcgggtatggtc |
| pRS423-1-ApCPS1-F | agttgaatattccctcaaaagtctctctctccctcagtggtcg |
| pRS423-1-ApCPS1-R | caaaaagagtctttgacttaatcagaagtaacggcgggtatgg |
| pRS423-1-GAL1.10p-F | tgatctcaagttgatggacattatagttttttctccttgacgttaaagtatagaggtat |
| pRS423-1-GAL1.10p-R | agaagaaagccatttatattgaattttcaaaaattcttactttttttttggatggacgc |
| pRS423-1-GALp-F | actcactatagggcgaattgtttgccagcttactatccttcttgaaaatatgc |
| pRS423-1-GALp-R | ctgagggagagagagacttttgagggaatattcaactgtttttttttatcatgttgatg |
| pRS423-1-KO-F | tttttgaaaattcaatataaatggctttcttctctatgatctctatcttgttg |
| pRS423-1-KO-R | tttaatttgcggccggtaccttatgatcttcttgggttgatgatagccattaatgg |
| pRS423-1-KS-F | atacccgccgttacttctgattaagtcaaagactctttttgcagagaaactgg |
| pRS423-1-KS-R | gtcaaggagaaaaaactataatgtccatcaacttgagatcatctggtt |
| pRS423-1-TEFt-F | catcaacccaagaagatcataaggtaccggccgcaaattaaagc |
| pRS423-1-TEFt-R | tcactaaagggaacaaaagctgctcgagtcatgtaattagttatgtcacgcttac |
| pRS423-2-ApCPS2-F | agttgaatattccctcaaaagtctctctctccctcagtggtcg |
| pRS423-2-ApCPS2-R | caaaaagagtctttgacttaatcagaagtaacggcgggtatgg |
| pRS423-2-GAL1.10p-F | tgatctcaagttgatggacattatagttttttctccttgacgttaaagtatagaggtat |
| pRS423-2-GAL1.10p-R | agaagaaagccatttatattgaattttcaaaaattcttactttttttttggatggacgc |
| pRS423-2-GALp-F | actcactatagggcgaattgtttgccagcttactatccttcttgaaaatatgc |
| pRS423-2-GALp-R | ctgagggagagagagacttttgagggaatattcaactgtttttttttatcatgttgatg |
| pRS423-2-KO-F | tttttgaaaattcaatataaatggctttcttctctatgatctctatcttgttg |
| pRS423-2-KO-R | tttaatttgcggccggtaccttatgatcttcttgggttgatgatagccattaatgg |
| pRS423-2-KS-F | atacccgccgttacttctgattaagtcaaagactctttttgcagagaaactgg |
| pRS423-2-KS-R | gtcaaggagaaaaaactataatgtccatcaacttgagatcatctggtt |
| pRS423-2-TEFt-F | catcaacccaagaagatcataaggtaccggccgcaaattaaagc |
| pRS423-2-TEFt-R | tcactaaagggaacaaaagctgctcgagtcatgtaattagttatgtcacgcttac |
| pRS423-3-ApCPS3-F | agttgaatattccctcaaaagtctctctctccctcagtggtcg |
| pRS423-3-ApCPS3-R | caaaaagagtctttgacttaatcagaagtaacggcgggtatgg |
| pRS423-3-GAL1.10p-F | tgatctcaagttgatggacattatagttttttctccttgacgttaaagtatagaggtat |
| pRS423-3-GAL1.10p-R | agaagaaagccatttatattgaattttcaaaaattcttactttttttttggatggacgc |
| pRS423-3-GALp-F | actcactatagggcgaattgtttgccagcttactatccttcttgaaaatatgc |
| pRS423-3-GALp-R | ctgagggagagagagacttttgagggaatattcaactgtttttttttatcatgttgatg |
| pRS423-3-KO-F | tttttgaaaattcaatataaatggctttcttctctatgatctctatcttgttg |
| pRS423-3-KO-R | tttaatttgcggccggtaccttatgatcttcttgggttgatgatagccattaatgg |
| pRS423-3-KS-F | atacccgccgttacttctgattaagtcaaagactctttttgcagagaaactgg |
| pRS423-3-KS-R | gtcaaggagaaaaaactataatgtccatcaacttgagatcatctggtt |
| pRS423-3-TEFt-F | catcaacccaagaagatcataaggtaccggccgcaaattaaagc |
| pRS423-3-TEFt-R | tcactaaagggaacaaaagctgctcgagtcatgtaattagttatgtcacgcttac |
| pRS423-4-ApCPS4-R | caaaaagagtctttgacttaatcagaagtaacggcgggtatgg |
| pRS423-4-GAL1.10p-F | tgatctcaagttgatggacattatagttttttctccttgacgttaaagtatagaggtat |
| pRS423-4-GAL1.10p-R | agaagaaagccatttatattgaattttcaaaaattcttactttttttttggatggacgc |
| pRS423-4-GALp-F | actcactatagggcgaattgtttgccagcttactatccttcttgaaaatatgc |
| pRS423-4-KO-F | tttttgaaaattcaatataaatggctttcttctctatgatctctatcttgttg |
| pRS423-4-KO-R | tttaatttgcggccggtaccttatgatcttcttgggttgatgatagccattaatgg |
| pRS423-4-KS-F | atacccgccgttacttctgattaagtcaaagactctttttgcagagaaactgg |
| pRS423-4-KS-R | gtcaaggagaaaaaactataatgtccatcaacttgagatcatctggtt |
| pRS423-4-TEFt-F | catcaacccaagaagatcataaggtaccggccgcaaattaaagc |
| pRS423-4-TEFt-R | tcactaaagggaacaaaagctgctcgagtcatgtaattagttatgtcacgcttac |
| pRS423-4-ApCPS4-F | agttgaatattccctcaaaaatgcacaaggtgcccacaacatta |
| pRS423-4-GALp-R | ggcaccttgtgcatttttgagggaatattcaactgtttttttttatcatgttgatg |
| pRS423-5-ApCPS5-F | agttgaatattccctcaaaagtctctctctccctcagtggtcg |
| pRS423-5-ApCPS5-R | caaaaagagtctttgacttaatcagaagtaacggcgggtatgg |
| pRS423-5-GAL1.10p-F | tgatctcaagttgatggacattatagttttttctccttgacgttaaagtatagaggtat |
| pRS423-5-GAL1.10p-R | agaagaaagccatttatattgaattttcaaaaattcttactttttttttggatggacgc |
| pRS423-5-GALp-F | actcactatagggcgaattgtttgccagcttactatccttcttgaaaatatgc |
| pRS423-5-GALp-R | ctgagggagagagagacttttgagggaatattcaactgtttttttttatcatgttgatg |
| pRS423-5-KO-F | tttttgaaaattcaatataaatggctttcttctctatgatctctatcttgttg |
| pRS423-5-KO-R | tttaatttgcggccggtaccttatgatcttcttgggttgatgatagccattaatgg |
| pRS423-5-KS-F | atacccgccgttacttctgattaagtcaaagactctttttgcagagaaactgg |
| pRS423-5-KS-R | gtcaaggagaaaaaactataatgtccatcaacttgagatcatctggtt |
| pRS423-5-TEFt-F | catcaacccaagaagatcataaggtaccggccgcaaattaaagc |
| pRS423-5-TEFt-R | tcactaaagggaacaaaagctgctcgagtcatgtaattagttatgtcacgcttac |
| pRS423-6-APS2-F | agttgaatattccctcaaaagtctctctctccctcagtggtcg |
| pRS423-6-APS2-R | tttaatttgcggccggtacctcagaagtaacggcgggtatgg |
| pRS423-6-GAL7p-F | gactcactatagggcgaattgtttgccagcttactatccttcttgaaaatatgc |
| pRS423-6-GAL7p-R | ctgagggagagagagacttttgagggaatattcaactgtttttttttatcatgttgatg |
| pRS423-6-TEF1t-F | atacccgccgttacttctgaggtaccggccgcaaattaaagc |
| pRS423-6-TEF1t-R | actaaagggaacaaaagctgctcgagtcatgtaattagttatgtcacgcttac |
| pRS423-7-ApCPS2-F | agttgaatattccctcaaaagtctctctctccctcagtggtcg |
| pRS423-7-ApCPS2-R | cgtcaaggagaaaaaactatatcagaagtaacggcgggtatgg |
| pRS423-7-GAL1.10p-F | atacccgccgttacttctgatatagttttttctccttgacgttaaagtatagaggtat |
| pRS423-7-GAL1.10p-R | aatgctatcccgcttatattgaattttcaaaaattcttactttttttttggatggacgc |
| pRS423-7-GALp-F | actcactatagggcgaattgtttgccagcttactatccttcttgaaaatatgc |
| pRS423-7-GALp-R | ctgagggagagagagacttttgagggaatattcaactgtttttttttatcatgttgatg |
| pRS423-7-KSL1-F | tttttgaaaattcaatataagcgggatagcattagtagagaagtgg |
| pRS423-7-KSL1-R | tttaatttgcggccggtacctctgaatatgcatttcatcgtgcagagc |
| pRS423-7-TEFt-F | cacgatgaaatgcatattcagaggtaccggccgcaaattaaagc |
| pRS423-7-TEFt-R | tcactaaagggaacaaaagctgctcgagtcatgtaattagttatgtcacgcttac |

**Supplementary Table S4 Information of similar functional genes in evolutionary tree.**

| Protein | Species | GenBank Accession | Annotation |
| --- | --- | --- | --- |
| **CPSs** | | | |
| *Sm*CPS_ent_ | *Salvia miltiorrhiza* | ALX18648 | *ent-*copalyl diphosphate synthase |
| *Zm*CPS | *Zea mays* | AAT70084 | *ent*-copalyl diphosphate synthase |
| *Ta*CPS | *Triticum aestivum* | BAH56558 | *ent-*copalyl diphosphate synthase |
| *Os*CPS | *Oryza sativa* | AAT11021 | *ent*-copalyl diphosphate synthase |
| *Ps*CPS | *Picea sitchensis* | ADB55709 | *ent*-copalyl diphosphate synthase |
| *Pg*CPS | *Picea glauca* | ADB55707 | *ent*-copalyl diphosphate synthase |
| *Ie*CPS | *Isodon eriocalyx* | AWN06649 | *ent*-copalyl diphosphate synthase |
| *Sd*CPS | *Salvia divinorum* | APH81399 | *ent*-copalyl diphosphate synthase |
| *Ac*CPS | *Aconitum carmichealii* | QWD59164 | *ent*-copalyl diphosphate synthase |
| *Cs*CPS | *Camellia sinensis* | QNN26117 | *ent*-copalyl diphosphate synthase |
| *Si*CPS | *Sesamum indicum* | UNZ93495 | *ent*-copalyl diphosphate synthase |
| *Ss*CPS | *Salvia splendens* | UNZ93492 | *ent*-copalyl diphosphate synthase |
| *Sb*CPS | *Scutellaria baicalensis* | UNZ93477 | *ent*-copalyl diphosphate synthase |
| *Lj*CPS | *Leonurus japonicus* | UVE15959 | *ent*-copalyl diphosphate synthase |
| *At*CPS | *Arabidopsis thaliana* | AAA53632 | *ent*-copalyl diphosphate synthase |
| *Cm*CPS | *Cucurbita maxima* | AAD04292 | *ent*-copalyl diphosphate synthase |

| Mutation | Mutation Energy | Effect of Mutation | VDW Term | Electrostatic Term | Entropy Term |
| --- | --- | --- | --- | --- | --- |
| A:ASP366>MET | -1.27 | STABILIZING | -1.35 | -1.23 | 0.02 |
| A:GLY452>TRP | -1.06 | STABILIZING | -2.18 | 0.14 | -0.05 |
| A:MET413>LEU | -1.05 | STABILIZING | -2.15 | 0.07 | -0.01 |
| A:ASP409>ILE | -1.02 | STABILIZING | -0.38 | -1.63 | -0.02 |
| A:MET413>SER | -0.99 | STABILIZING | -2.02 | -0.03 | 0.04 |
| A:ASP366>ASN | -0.98 | STABILIZING | -0.95 | -1.01 | 0 |
| A:GLY537>THR | -0.98 | STABILIZING | -2.02 | 0.13 | -0.04 |
| A:ALA412>TYR | -0.96 | STABILIZING | -1.85 | -0.06 | 0 |
| A:MET413>ASN | -0.94 | STABILIZING | -2.44 | 0.57 | 0 |
| A:CYS441>ARG | -0.94 | STABILIZING | -1.9 | 0.05 | -0.02 |
| A:GLY537>GLN | -0.92 | STABILIZING | -1.96 | 0.16 | -0.03 |
| A:ASP366>THR | -0.91 | STABILIZING | -0.75 | -1.08 | 0.01 |
| A:GLY452>PHE | -0.9 | STABILIZING | -1.84 | 0.11 | -0.04 |

**Supplementary Table S5 *Ap*CPS2 saturated mutation sites.**

**Supplementary Figures**


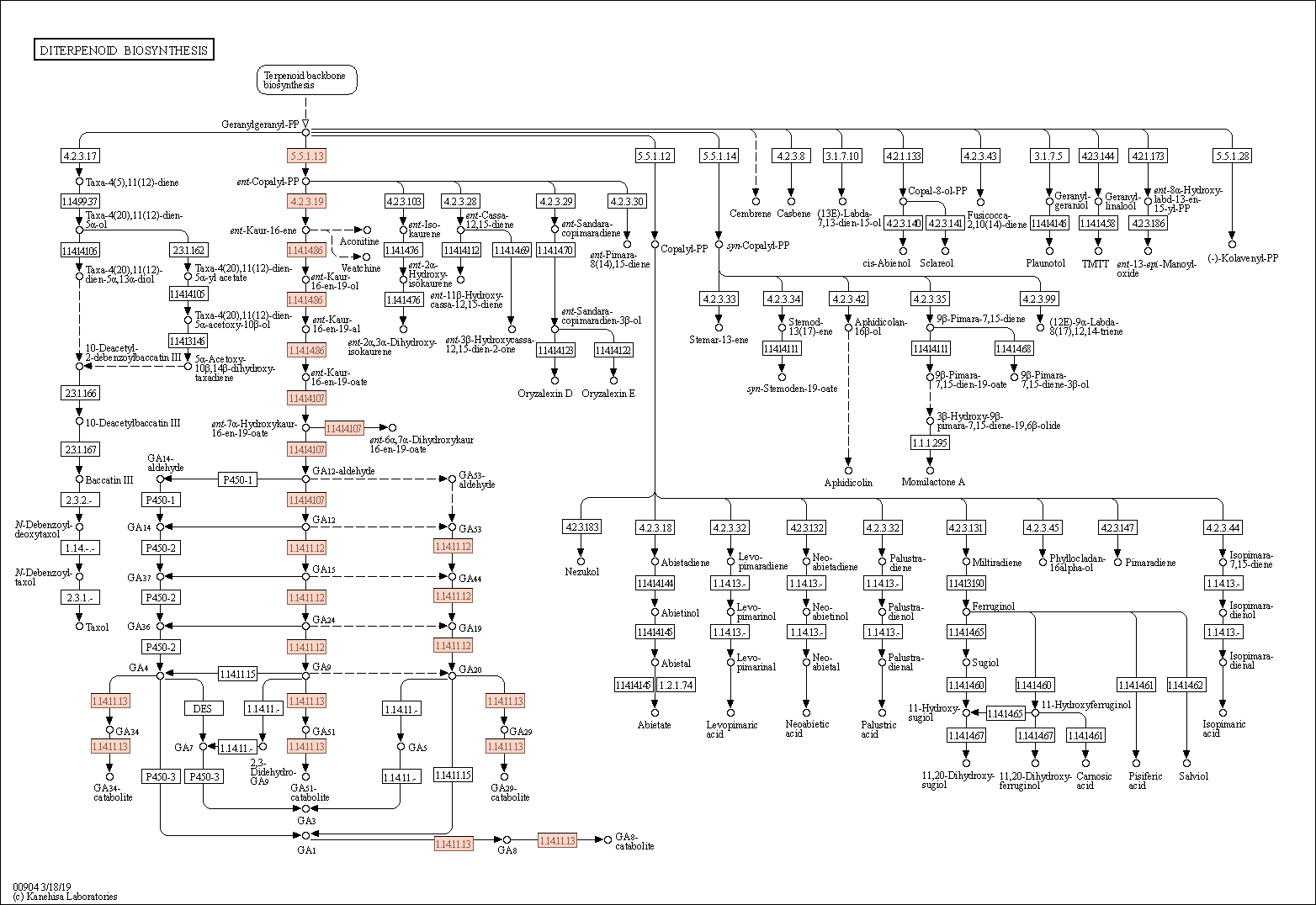


**Supplementary Figure S1. Diterpenoid network metabolism diagram of *Andrographis paniculata*.**


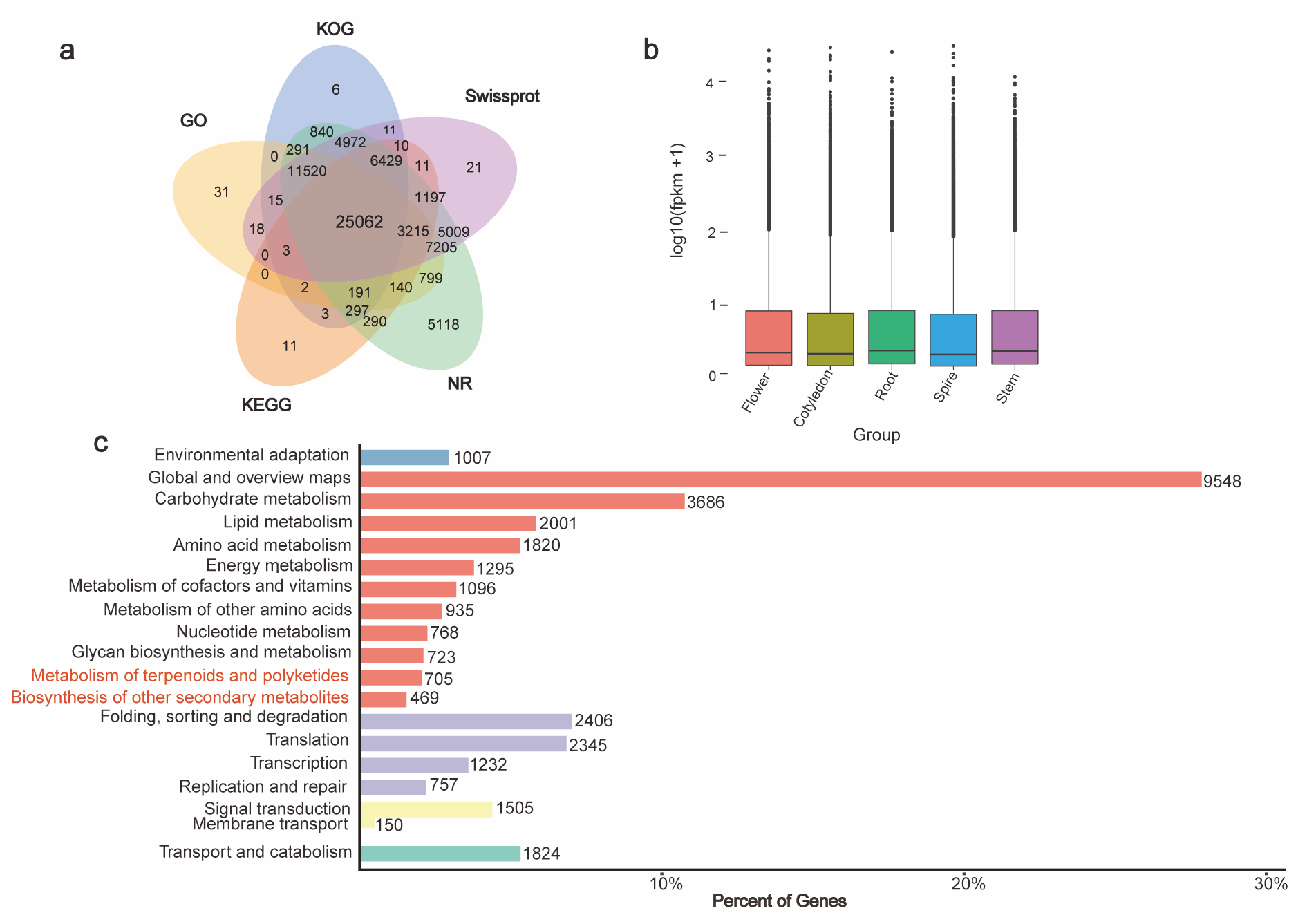


**Supplementary Figure S2. Transcriptomic analysis of *A. paniculata*.**

(**a**) Gene annotation profile of obtained from five databases. (**b**) Distribution of FPKM values of genes from different *A. paniculata* tissues. (**c**) Distribution of candidate genes among functional categories.


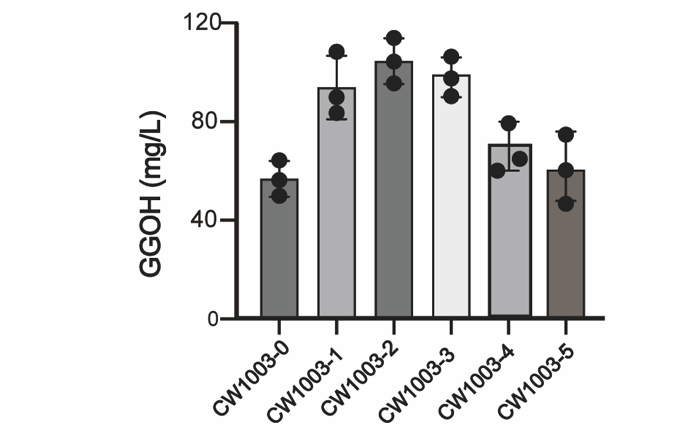


**Supplementary Figure S3. GGPP diterpene synthase screening.**


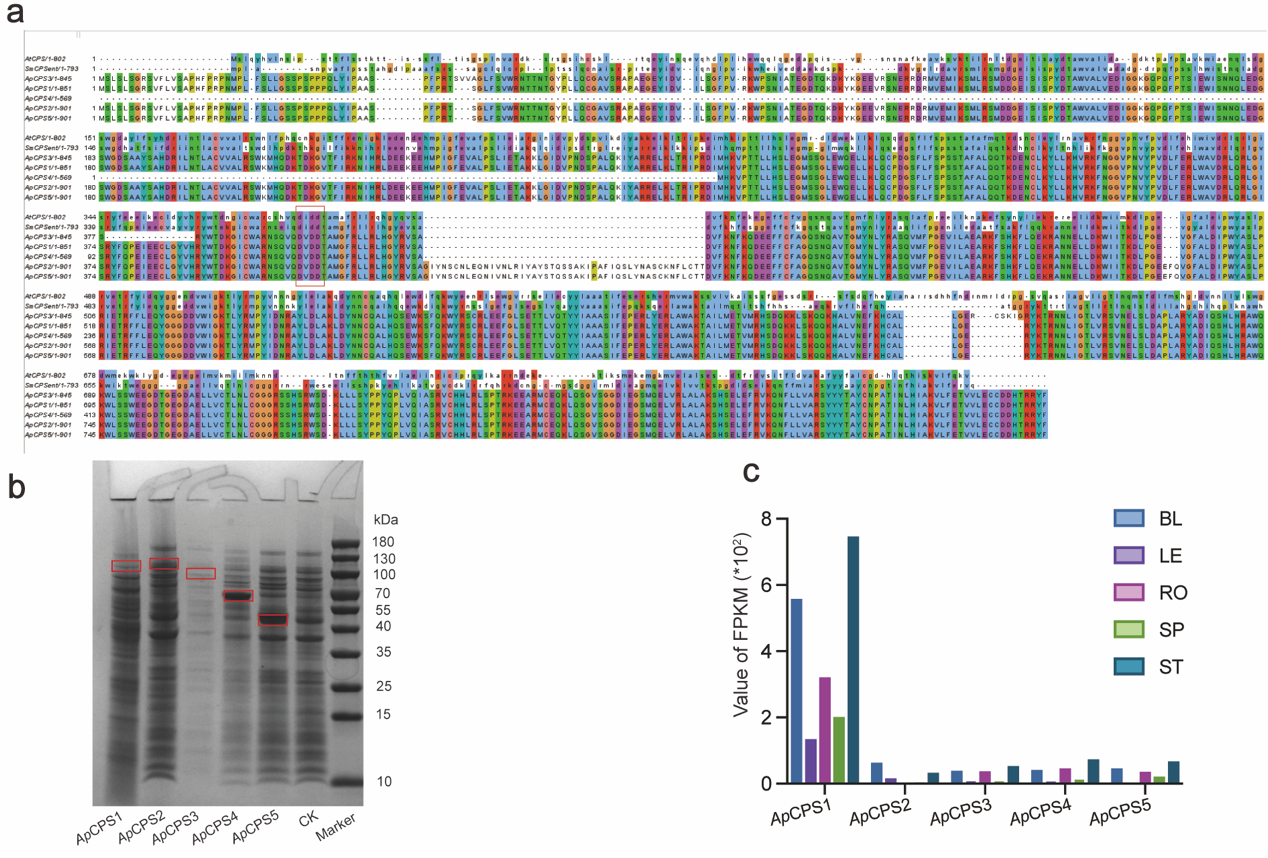


**Supplementary Figure S4. Sequence and expression verification of *Ap*CPS1-5.**

(a) Sequence comparison between *Ap*CPS1-5 and *Sm*CPS; (b) SDS-PAGE of *Ap*CPS1-5 protein expression; (c) Expression of ApCPS1-5 in different tissues.

*Ap*CPS1-102 kDa；*Ap*CPS2-108 kDa；*Ap*CPS3-101 kDa；*Ap*CPS4-68 kDa；*Ap*CPS5-53 kDa; BL-bloom; LE-leaf; RO-root; Sp-spire; ST-stem.
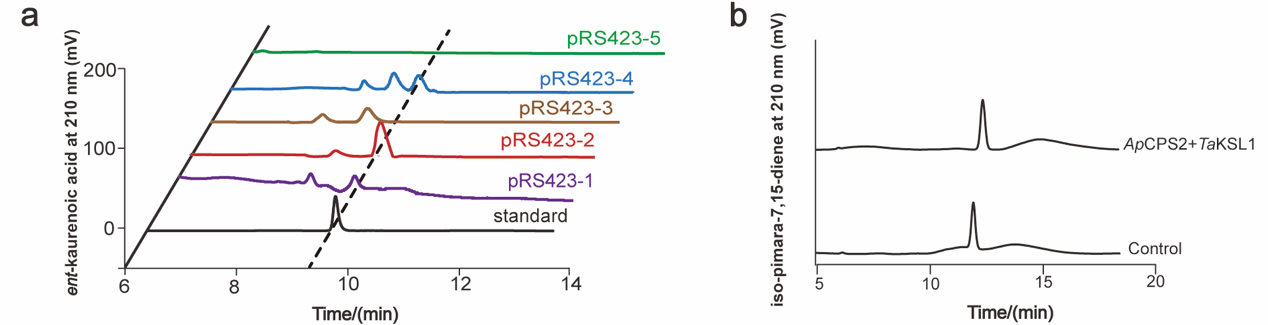


**Supplementary Figure S5. Product configuration verification of *Ap*CPS1-5.**

(a) *Ap*CPS1-5 produces *ent*-kaurenoic acid; (b) LC diagram of *Ap*CPS2 and *Ta*KSL1 reaction.


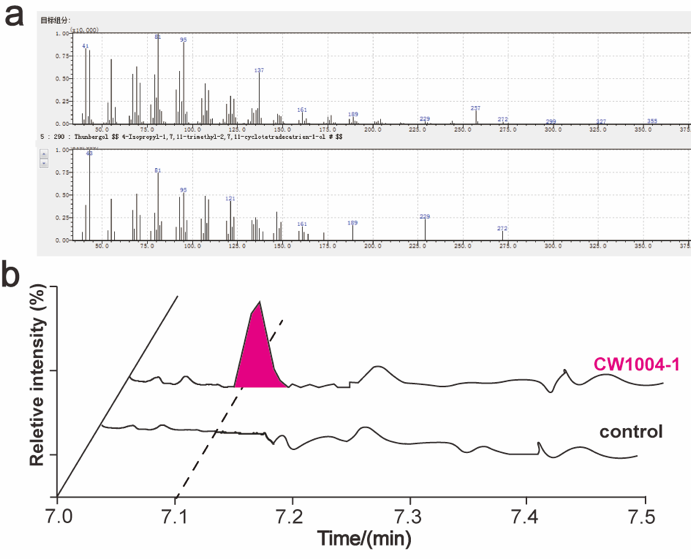


**Supplementary Figure S6. The fermentation product was detected by GC-MS.**

(a) GC-MS shows the ionic fragment of *ent*-copalol. (b) *ent*-Copalol peak of the CW1004-1 culture broth.


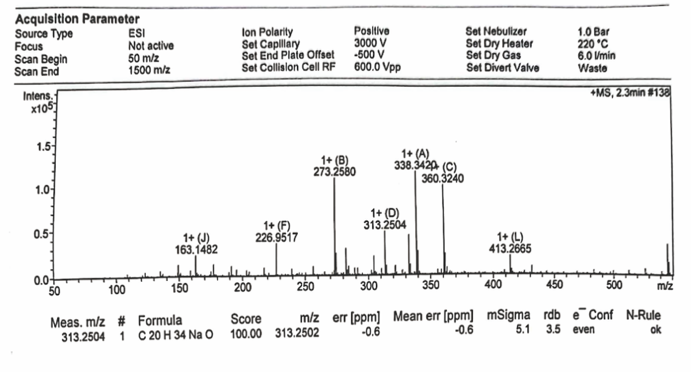


**Supplementary Figure S7. ESI-MS peak of substance with *ent*-copalol.**


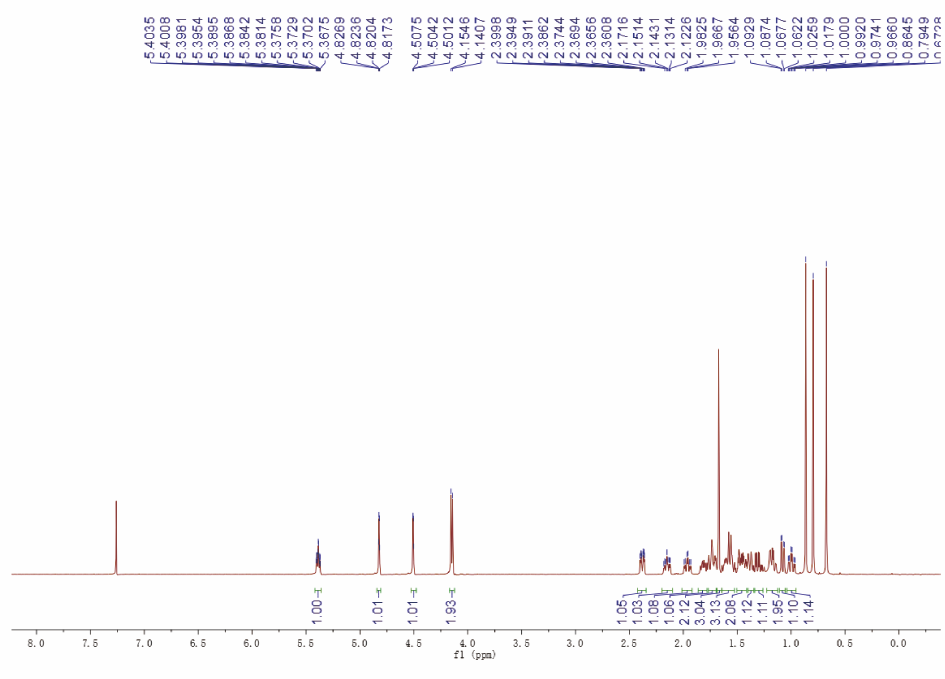


**Supplementary Figure S8. ^1^H NMR spectrum of *ent*-copalol (500 MHz, CDCl3).**


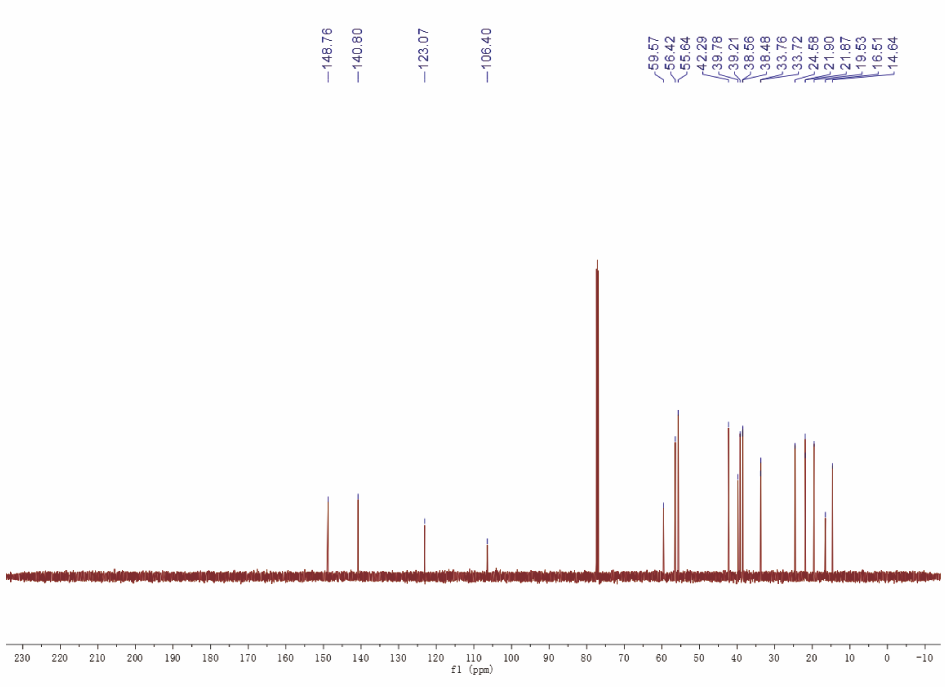


**Supplementary Figure S9. ^13^C NMR spectrum of *ent*-copalol (126 MHz, CDCl3).**

**NMR data for *ent*-copalol**

^1^H NMR (500 MHz, CDCl3 δ 5.39 (tq, *J* = 6.9, 1.3 Hz, 1H), 4.82 (q, *J* = 1.6 Hz, 1H), 4.50 (t, *J* = 1.6 Hz, 1H), 4.15 (d, *J* = 7.0 Hz, 2H), 2.38 (ddd, *J* = 12.7, 4.3, 2.4 Hz, 1H), 2.15 (ddd, *J* = 14.4, 10.2, 4.4 Hz, 1H), 1.96 (td, *J* = 13.0, 5.2 Hz, 1H), 1.86 – 1.79 (m, 1H), 1.79 – 1.69 (m, 2H), 1.67 (s, 3H), 1.65 – 1.51 (m, 3H), 1.51 – 1.43 (m, 2H), 1.42 – 1.36 (m, 1H), 1.31 (qd, *J* = 13.0, 4.3 Hz, 1H), 1.17 (td, *J* = 13.3, 4.1 Hz, 1H), 1.08 (dd, *J* = 12.6, 2.8 Hz, 1H), 1.00 (td, *J* = 13.0, 4.0 Hz, 1H), 0.86 (s, 3H), 0.79 (s, 3H), 0.67 (s, 3H).

^13^C NMR (126 MHz, CDCl_3_) δ 148.8, 140.8, 123.1, 106.4, 59.6, 56.4, 55.6, 42.3, 39.8, 39.2, 38.6, 38.5, 33.8, 33.7, 24.6, 21.9, 21.9, 19.5, 16.5, 14.6.
